# Transmission line faults detection and classification using new tripping characteristics based on statistical coherence for current measurements

**DOI:** 10.1038/s41598-025-87577-5

**Published:** 2025-03-12

**Authors:** R. A. Mahmoud

**Affiliations:** Department of Electrical Power and Machines Engineering (PME), College of Engineering Science and Technology, Misr University for Science and Technology (MUST), 6th of October City, Giza, Egypt

**Keywords:** Power transmission line, Shunt faults, Series faults, Fault detection, Fault classification, Coherence method, Energy science and technology, Engineering

## Abstract

Power transmission lines are critical components of a power system that connect power stations to consumers. To maintain reliability and stability of the system, faults should be correctly classified and cleared as soon as possible. In this article, a coherence-based protection scheme for faults detection and classification on transmission lines (TLs) is proposed. Besides, the scheme introduces a new model of tripping characteristics based on six coherence coefficients that are computed only for current waves measured at the TL sending end. The power network under test is simulated using the ATP software, and signals analysis and the performance evaluation of the technique are performed in the MATLAB environment. The protection performance is investigated under different fault conditions, such as fault type, fault location, fault resistance, fault inception angle and power flow angle. The extensive simulation cases have demonstrated that the suggested technique is successful in detecting and classifying all ten shunt faults in the transmission line within a half-cycle time. Moreover, the protection security, sensitivity, and response speed are amended by changing the numerical values of the coherence setting and data window. Furthermore, it is applicable in both conventional and smart grids, and it is independent of the specifications of the system equipment and current transformers.

## Introduction

Power transmission lines (TLs) are vital elements of power systems because they link generating stations to consumers in order to achieve a power supply continuity^1^. Moreover, it connects between the systems for bidirectional power flow. Large blocks of electrical power can be economically transferred between these systems with high-voltage TLs^2^. It is important to have a protection system against TL faults in the electrical networks because most faults occur on TL^3^. TL faults can be divided into three types: shunt unsymmetrical, shunt symmetrical and series faults^4^. There are three forms of the unsymmetrical shunt faults (i.e., SLG, DL, and DLG), and one form of the symmetrical shunt faults (i.e., 3 L or 3LG)^5^. Such transient faults can cause overvoltage and/or overcurrent, which can damage insulation materials and conductors of the power system depending upon their severity^6^. Furthermore, they contain useful information that can be used for analyzing TL disturbances^7^. It is essential to detect and locate the faults quickly and to classify them correctly in order to maintain the system stability and reliability, and to provide high energy quality^8^. TL protective relays always utilize three-phase currents, voltages or both to perform the task of fault detection, faulty phase(s) identification, classification, and location^9^. The protective relays send a tripping control signal in order to TL CB(s) to isolate the faulted TL from the rest of the system^10^. Traditional distance relays are the primary protection of TLs, which estimate the impedance between the relay and the fault locations, and respond to the distance to the TL fault^11^. The relay speed, reliability and accuracy of the fault detection and classification are important protection requirements for the lines. With the applications of signals processing and modern techniques, a TL protection development for fault diagnosis has been progressed^12^. Various techniques of fault detection, fault classification, fault location, faulty phase(s) selection, and fault direction discrimination were approached in several articles. Some techniques used synchro-phasors^1^, Travelling Waves (TW)^2,3^, least error squares^4^, sequential components^5,6^, a combination of neural network and PMU measurements^7^, a combination of Wavelet and alienation^8^, genetic algorithm^9^, signal processing^10^, and statistical methods such as alienation^11^, correlation^12^, and phasor measurement unit (PMU)^13^. Moreover, other methods were based on the S-transform^14^, Discrete Fourier Transform (DFT)^15^, voltage Travelling Waves (TW)^16^, Wavelet Packet Transform (WPT)^17^, Support Vector Machine (SVM)^18^, machine learning^19,20^, fuzzy logic^21^, Decision Tree (DT)^22^, differential protection based on an incremental complex power alpha plane^23^, and differential protection based on Continuous Wavelet Transform (CWT)^24^.

Several techniques for TL fault identification were also presented, including single-ended travelling wave^25^, travelling wave theory^26^, decision tree aided travelling wave^27^, voltage response^28^, transmission line topology, non-unit transient^29^, source impedance^30^, and the pilot impedance supported by synchronized data^31^. The technique in^32^ suggested an integrated method for discriminating internal and external faults and locating the faults on the TL compensated with a unified power flow controller. The paper^33^ introduced a digital protection based on entropy principle, fast discrete orthogonal S-transform, support vector machine, and support vector regression for faults detection, classification, and location on hybrid TL consisting of Overhead Transmission Line (OHTL) and Underground Cable (UGC) sections. The method reported in^34^ was dependent on a Gaussian Naïve Bayes approach for TL fault classification using phasor measurements. A modification of state estimation formulation was used in^35^ for the fault location, selection of faulty line, identification of faulted phase(s), and fault classification in the power network over a wide-area. TL fault determination, classification, and location were also verified using travelling wave frequencies and extreme learning machine^36^, wide area measurement^37^, Gabor transform^38^, and polarity and arrival time of asynchronously recorded travelling wave^39^.

Additionally, several transmission line protection solutions were recently published, including those based on distances between current samples using the Hausdorff Distance^40^, Euclidean Distance^41^, Canberra Distance^42^, and Dynamic Time Warping^43^. In addition, other protections were contingent on statistical techniques using the Pearson Correlation^44^, Spearman Correlation^45^, Kendall Tau Correlation^46^, Biweight Mid-correlation^47^, Dice Coefficient^48^, Bayesian Inference^49^, Structural Similarity^50^, Cosine Similarity^51,52^, and an impedance estimation–based protection using Lissajous Fig^53^.

In this paper, a fault detection, classification, and faulty phase(s) identification algorithm is provided as a secondary protection for TL primary protections using a coherence function. This algorithm relies on three auto-coherence and three cross-coherence coefficients to identify the fault occurrence, discriminate the faulty phase(s), and classify the ten types of shunt faults. Three-phase current waves are used at only one end to process the proposed protection algorithm. Extensive simulation cases are conducted on a typical power system to verify the algorithm’s performance. All fault types, locations, inception angles and fault impedance will be included in this study. The results demonstrate that the proposed algorithm provides efficient discrimination between all fault types within a sub-cycle time, increases protection requirements such as robustness, security and dependability, and makes an accurate decision when faced with the faults. In the present manuscript, the contributions to knowledge are given as follows:

1. A new protection scheme is proposed to detect, select the faulty phase(s), and classify the different types of faults using the auto- and cross-coherence functions computed for only current waves measured at the TL sending end,
2. The digital protection scheme has used two integrated modules to detect and classify the different TL faults, as well as to select the faulty phase(s),
3. Only two types of mathematical equations are applied for employing the protection algorithm. For the current signals, three auto-coherence factors and three cross-coherence factors can be calculated using these equations.
4. The method introduces new models of tripping curves based on the auto- and cross-coherence coefficients quantified for three-phase current signals. It can be used to isolate the faulty system, while keeping the power grid safe during the normal operation,
5. The approach can control the operating time required to identify the fault quickly by changing the selected data set. One-cycle or sub-cycle time can be chosen, and.
6. Only one coherence setting can be selected to discriminate between the normal and abnormal states, as well as to achieve a compromise between the relay sensitivity and security levels.

The article is organized as follows: In Sect. 2, the coherence-based protection algorithm is discussed. In Sect. 3, a typical power system model with a list of real data parameters is demonstrated. Section 4 presents the results of various case studies, as well as an analysis of the technique performance for fault detection and classification. Advanced technique properties and a comparison between the proposed and published protection techniques are demonstrated in Sect. 5. Ultimately, the conclusions are drawn in Sect. 6.

## Protection methodology

### Coherence application justification

The proposed TL protection is based on recent advances in signal processing and numerical analysis for discrete values of the three-phase currents measured at only one end of the protected transmission line. It is capable of detecting the fault occurrence, selecting the faulty phase(s) and classifying the different types of the TL faults using the coherence function. There are two types of coherence: auto-coherence and cross-coherence^54–56^. The coherence factors in this study are divided into three auto-coherence factors and three cross-coherence factors^55–57^.

The coherence function possesses the following attributes:

1. Creating new, square-shaped relay tripping curves that are bounded between the two real values of 0.0 and + 1.0,
2. Distinguishing between the healthy and faulty states for the power TLs using both auto-coherence and cross-coherence functions. The auto-coherence factors obtained for the three-phase currents are constant and equal to approximately + 1.0 in the normal operating conditions, while they are unstable and unequal in fault states. Also, the cross-coherence factor calculated for each two phase currents is fixed and close to + 0.25 in the healthy conditions, while the three cross-coherence factors are upset and unlike in fault events,
3. Selecting the faulty phase(s), and classifying the ten types of shunt faults using the auto-coherence function. The relationship is strong and stable when the auto-coherence factor computed for each phase current is nearly + 1.0, while it is weak and unstable when the auto-coherence factor is otherwise less than + 1.0, and.
4. Differentiating between the DL and DLG faults using the cross-coherence function. The cross-coherence factor calculated for the two currents of the faulty phases is equal to about + 1.0 in the case of the DL fault, while its value is disturbed and unequal to unity in the case of the DLG fault.

### Coherence mathematical formulas

#### Auto-coherence function

The auto-coherence coefficient (*Ci*_*s*_(*k*)) can be found between two data sets that are shifted by one-cycle time of the current wave for *‘S’* phase. This coefficient can be quantified using the following mathematical formula for each phase current^58,59^:

1 $$Ci_{s} (k) = \frac{{\left[ {\left( {\sum\limits_{{n = 0}}^{{N - 1}} {I_{{s1}} (k) \times I_{{s3}} (k) + I_{{s2}} (k) \times I_{{s4}} (k)} } \right)^{2} + \left( {\sum\limits_{{n = 0}}^{{N - 1}} {I_{{s1}} (k) \times I_{{s4}} (k) - I_{{s2}} (k) \times I_{{s3}} (k)} } \right)^{2} } \right]}}{{\sum\limits_{{n = 0}}^{{N - 1}} {\left[ {\left( {I_{{s1}} (k)} \right)^{2} + \left( {I_{{s2}} (k)} \right)^{2} } \right] \times \sum\limits_{{n = 0}}^{{N - 1}} {\left[ {\left( {I_{{s3}} (k)} \right)^{2} + \left( {I_{{s4}} (k)} \right)^{2} } \right]} } }}$$

Where,

$$I_{{s1}} (k) = \sum\limits_{{n = 0}}^{{N - 1}} {\left[ {i_{s} (n) \cdot \cos \left( {\frac{{2\pi kn}}{N}} \right)} \right]}$$

$$I_{{s2}} (k) = \sum\limits_{{n = 0}}^{{N - 1}} {\left[ {i_{s} (n) \cdot \sin \left( {\frac{{2\pi kn}}{N}} \right)} \right]}$$

$$I_{{s3}} (k) = \sum\limits_{{n = 0}}^{{N - 1}} {\left[ {i_{s} (n - N_{s} ) \cdot \cos \left( {\frac{{2\pi kn}}{N}} \right)} \right]}$$

$$I_{{s4}} (k) = \sum\limits_{{n = 0}}^{{N - 1}} {\left[ {i_{s} (n - N_{s} ) \cdot \sin \left( {\frac{{2\pi kn}}{N}} \right)} \right]}$$

##### Is1(k)

Cosine component of the DFT for wave *i*_*s*_*(n)*,

##### Is2(k)

Sine component of the DFT for wave *i*_*s*_*(n)*,

##### Is3(k)

Cosine component of the DFT for wave *i*_*s*_*(n-N*_*s*_*)*, and.

##### Is4(k)

Sine component of the DFT for wave *i*_*s*_*(n-N*_*s*_*).*

Equation (1) can be used to get the three auto-coherence coefficients: *Ci*_*a*_(*k*), *Ci*_*b*_(*k*), and *Ci*_*c*_(*k*).

#### Cross-coherence function

The cross-coherence coefficient (*Ci*_*sx*_(*k*)) can be determined between each two corresponding data sets of the two current waves (*i*_*s*_*(n)* and *i*_*x*_*(n)*) for the *‘S’* and *‘X’* phases. The following equation can be used to calculate the coefficient for each two phase currents^57–59^:

2 $$Ci_{{sx}} (k) = \frac{{\left[ {\left( {\sum\limits_{{n = 0}}^{{N - 1}} {I_{{s1}} (k) \times I_{{x1}} (k) + I_{{s2}} (k) \times I_{{x2}} (k)} } \right)^{2} + \left( {\sum\limits_{{n = 0}}^{{N - 1}} {I_{{s1}} (k) \times I_{{x2}} (k) - I_{{s2}} (k) \times I_{{x1}} (k)} } \right)^{2} } \right]}}{{\sum\limits_{{n = 0}}^{{N - 1}} {\left[ {\left( {I_{{s1}} (k)} \right)^{2} + \left( {I_{{s2}} (k)} \right)^{2} } \right] \times \sum\limits_{{n = 0}}^{{N - 1}} {\left[ {\left( {I_{{x1}} (k)} \right)^{2} + \left( {I_{{x2}} (k)} \right)^{2} } \right]} } }}$$

Where,

$$I_{{x1}} (k) = \sum\limits_{{n = 0}}^{{N - 1}} {\left[ {i_{x} (n) \cdot \cos \left( {\frac{{2\pi kn}}{N}} \right)} \right]}$$

$$I_{{x2}} (k) = \sum\limits_{{n = 0}}^{{N - 1}} {\left[ {i_{x} (n) \cdot \sin \left( {\frac{{2\pi kn}}{N}} \right)} \right]}$$

##### Ix1(k)

Cosine component of the DFT for wave *i*_*x*_*(n)*, and.

##### Ix2(k)

Sine component of the DFT for wave *i*_*x*_*(n).*

Equation (2) can be used to obtain the three cross-coherence coefficients: *Ci*_*ab*_(*k*), *Ci*_*bc*_(*k*), and *Ci*_*ca.*_(*k*). The concept of changing the amount of data window can be applied to the present algorithm. This study uses a pre-determined fixed value for the data window, which is selected to be a half-cycle time of the fundamental power frequency in the system.

### Proposed algorithm strategy

Two modules of the proposed protection methodology are sequentially processed as follows:

1. Module 1: It can be used to detect the fault occurrence, discriminate the faulty phase(s), and classify the fault types using the auto-coherence coefficients of the current waves, and.
2. Module 2: It can be used to ensure the fault presence, and distinguish between the DL and DLG faults using the cross-coherence factors of the current waves.

**Fig. 1:**
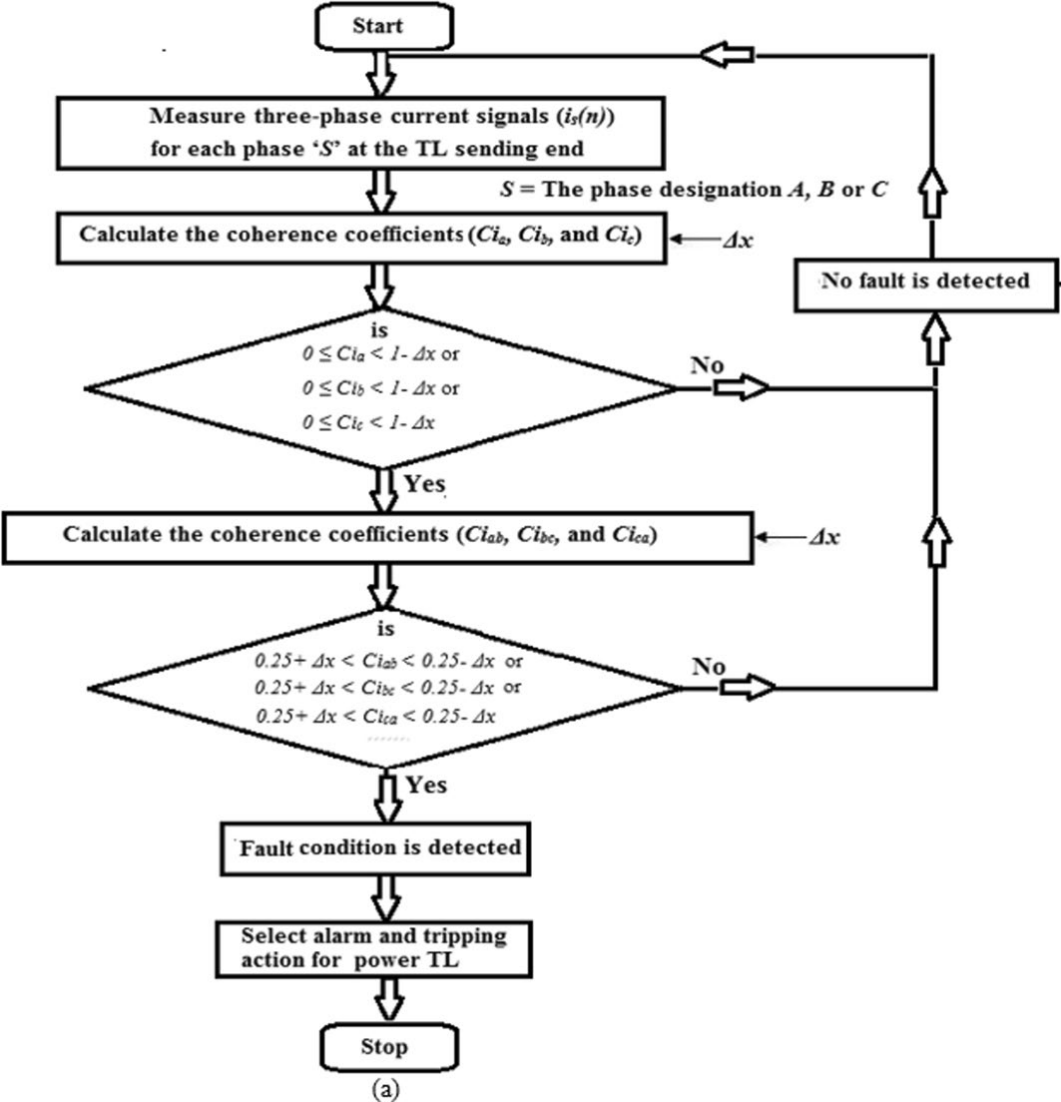

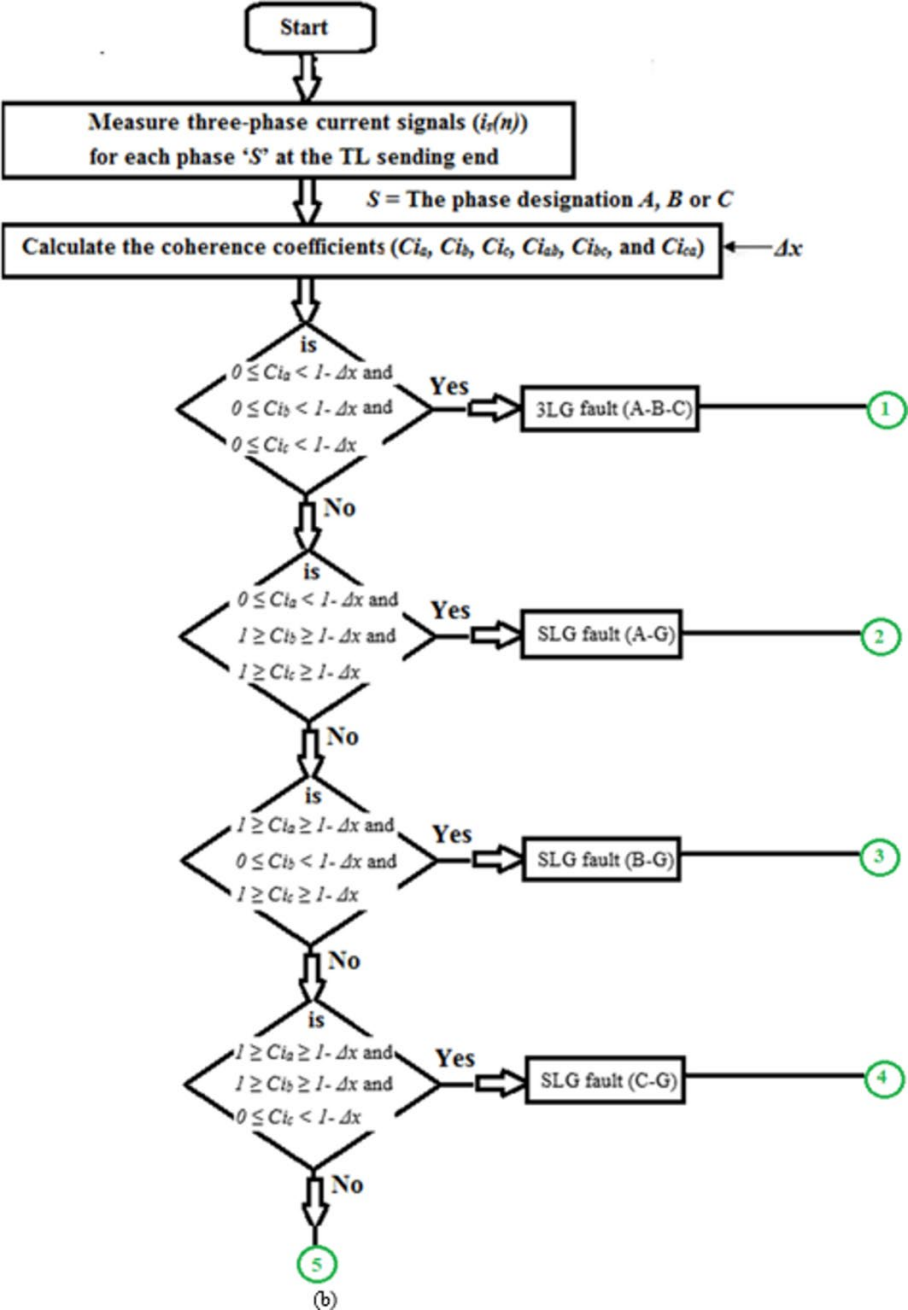

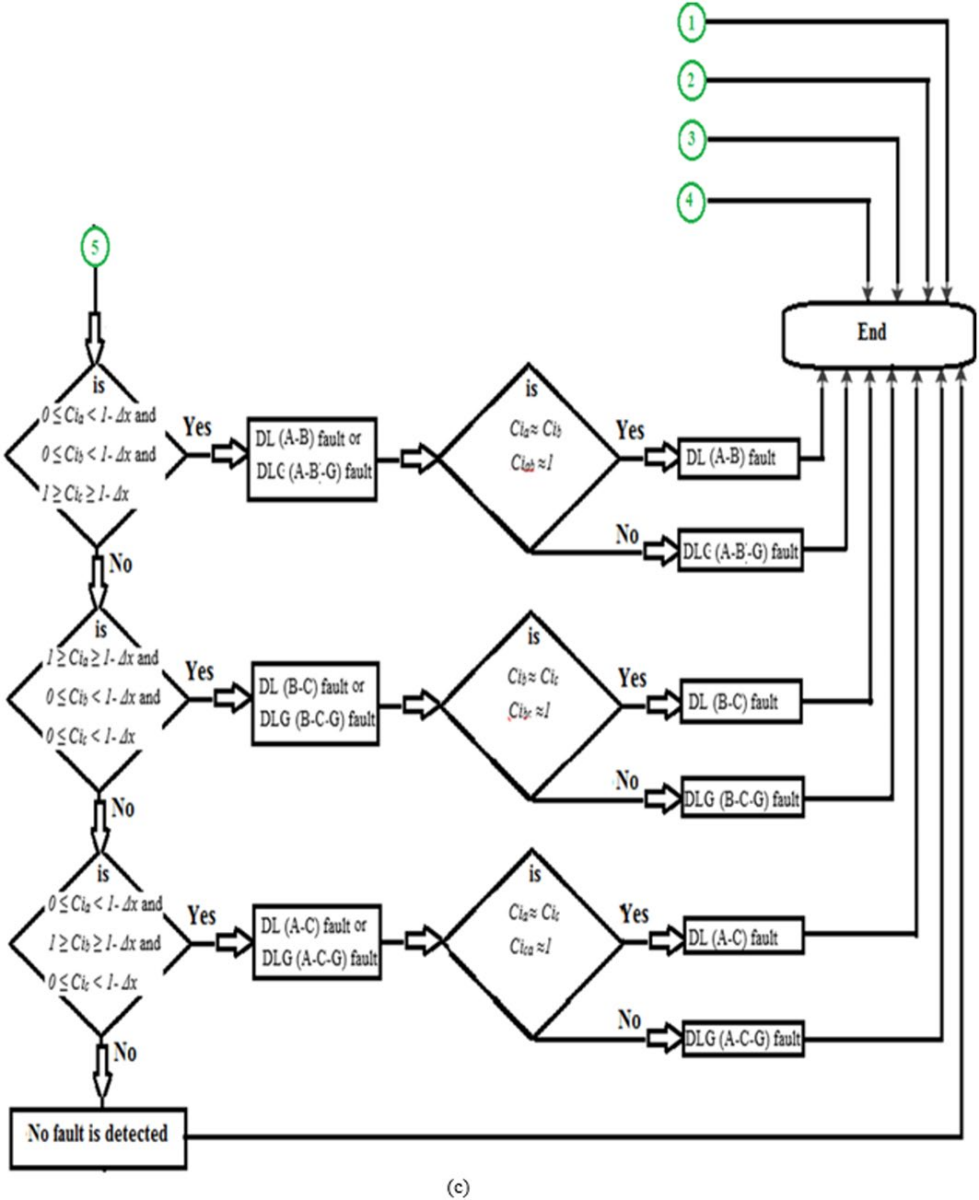
(**a**) Flow chart of the proposed method for fault detection. (**b**) Flow chart of the proposed method for fault classification. (**c**) Flow chart of the proposed method for fault classification (Continued).

Figure 1a presents the first flow chart of the proposed method for detecting fault events. Whereas, Fig. 1b, c show the second flow chart of the proposed method for classifying the ten shunt faults on the power transmission line. The proposed algorithm employs both auto-coherence and cross-coherence functions.

#### Fault detection

There are several variables that change when the TL fault occurs, including electrical voltage, current, power factor, impedance, and frequency. In addition, the parameters of power quality, such as magnitude, frequency, symmetry, and shape of the measured waveforms may be disturbed. The proposed technique can be used to determine the fault occurrence in three scenarios:

(I) The estimation of the auto-coherence factor for each phase current,
(II) The post-fault auto-coherence value of the phase current is compared with the pre-fault value for the same phase current, and.
(III) The values of the three auto-coherence factors are compared over the same time span.

In this algorithm, a fault transition can be detected if *0 ≤ Ci*_*a*_*˂* (*1- Δx*), *0 ≤ Ci*_*b*_*˂ (1- Δx*), or *0 ≤ Ci*_*c*_*˂* (*1- Δx*); where, the selected value of the coherence setting margin (*Δx*) is + 0.15.

#### Fault confirmation

To confirm that the fault is present, the following conditions should be satisfied:

(0.25 + Δx) **<** Ci_ab_**<** (0.25- Δx), (0.25 + Δx) **<** Ci_bc_**<** (0.25- Δx), or (0.25 + Δx) **<** Ci_ca._**<** (0.25- Δx).

#### Fault classification

After detecting the fault, fault classification and the faulty phase(s) discrimination can be accomplished according to the rules tabulated in (Table 1).

### Algorithm procedure

The sequence of the algorithm is as follows:

1. Read the instantaneous values of the three-phase currents (*i*_*s*_*(n)*) measured at the TL sending end,
2. Choose the numerical values for the data window area (*N*) and the coherence margin (*Δx*),
3. Quantify the three auto-coherence factors (*Ci*_*a*_, *Ci*_*b*_, and *Ci*_*c*_) using Eq. (1).
4. Differentiate between the healthy and the faulty states according to the conditions given in Table 1, and the flow chart depicted in (Fig. 1a). When the values of the three auto-coherence factors are within the range of + 1.0 and 1.0 *- Δx*, then the system status is healthy, while if the values of at least one factor are outside this range, then the system status is faulty.
5. Calculate the three cross-coherence factors (*Ci*_*ab*_, *Ci*_*bc*_, and *Ci*_*ca.*_) using Eq. (2).
6. Check the power system status is whether normal or abnormal according to the conditions in Table 1 and the flow chart shown in (Fig. 1a). If the values of the three cross-coherence factors are within the range of 0.*25 ± Δx*, then the system status is healthy, while if the values of at least one factor are outside this range, then the system status is faulty.
7. Discriminate the phases that are faulty, and classify the TL fault type according to the conditions given in (Table 1) and the flow chart depicted in (Fig. 1b).
8. The protection action is based on the conditions noted in (Table 1).

There are six coherence factors that can recognize fault conditions in the system, discriminate the faulty phase(s), classify the fault kind, and operate in response to it. The proposed algorithm monitors fault events continuously and sends a trip control signal to the TL CB(s) when there is a short-circuit current, while it will be held under normal operating conditions.

This scheme should take into account the following concerns:

(I) It is recommended that the setting margin (*Δx*) should be in between the two values of 0.0 and + 0.25,
(II) The number of samples per data set (*N*) should be lower than or equal to the number of samples per cycle (*N*_*s*_). This allows the proposed method to respond rapidly to detect short-circuit currents, and
(III) Changes to the protection security, sensitivity, and response speed can be made via the data set (*N*) and the coherence margin (*Δx*).

In this work, the auto-coherence function has the following roles:

(I) Detecting the fault situation,
(II) Selecting the faulty phase(s), and
(III) Classifying the fault type,

Whereas, the cross-coherence function has the following tasks:

(I) Confirming the fault event,
(II) Identifying and estimating the three-phase current imbalance, and
(III) Discriminating between DL and DLG faults.

When the auto-coherence factors assure that two phases are faulted, and the cross-coherence factor estimated between the two current waves of the two faulty phases is nearly + 1.0, the fault type is a DL fault. Otherwise, it is DLG.

**Table 1:** TL shunt faults detection and classification based on the six coherence factors computed for the current waves and the technique action.

Module type	Required signals	Coherence coefficients range	TL fault detection and classification	Technique action (tripping/blocking)
	The selected numerical value of the coherence margin (*Δx*) is *0.15*			
Module (1) for detecting the fault occurrence, selecting the faulty phase(s), and classifying the fault types using the auto-coherence coefficients for the current waves	*i*_*a*_*(n)* and *i*_*a*_*(n - N*_*s*_*)*	*1 ≥ Ci*_*a*_*≥ 1- Δx*, *1 ≥ Ci*_*b*_*≥ 1- Δx* and *1 ≥ Ci*_*c*_ *≥ 1- Δx*	Normal operation	Blocking
	*i*_*b*_*(n)* and *i*_*b*_*(n - N*_*s*_*)*			
	*i*_*c*_*(n)* and *i*_*c*_*(n - N*_*s*_*)*			
	*i*_*a*_*(n)* and *i*_*a*_*(n - N*_*s*_*)*	*0 ≤ Ci*_*a*_* <1- Δx*, *0 ≤ Ci*_*b*_* <1- Δx* or *0 ≤ Ci*_*c*_ *<1- Δx*	Fault detection	Tripping
	*i*_*b*_*(n)* and *i*_*b*_*(n - N*_*s*_*)*			
	*i*_*c*_*(n)* and *i*_*c*_*(n - N*_*s*_*)*			
	*i*_*a*_*(n)* and *i*_*a*_*(n - N*_*s*_*)*	*0 ≤ Ci*_*a*_* <1- Δx*, *0 ≤ Ci*_*b*_* <1- Δx*, *0 ≤ Ci*_*c*_* <1- Δx* and *Ci*_*a*_ ≈ *Ci*_*b*_ ≈ *Ci*_*c*_	3LG fault (A-B-C-G) or 3 L fault (A-B-C)	Tripping
	*i*_*b*_*(n)* and *i*_*b*_*(n - N*_*s*_*)*			
	*i*_*c*_*(n)* and *i*_*c*_*(n - N*_*s*_*)*			
	*i*_*a*_*(n)* and *i*_*a*_*(n - N*_*s*_*)*	*0 ≤ Ci*_*a*_* <1- Δx*, *1 ≥ Ci*_*b*_*≥ 1- Δx* and *1 ≥ Ci*_*c*_ *≥ 1- Δx*	SLG fault (A-G)	Tripping
	*i*_*b*_*(n)* and *i*_*b*_*(n - N*_*s*_*)*			
	*i*_*c*_*(n)* and *i*_*c*_*(n - N*_*s*_*)*			
	*i*_*a*_*(n)* and *i*_*a*_*(n - N*_*s*_*)*	*1 ≥ Ci*_*a*_*≥ 1- Δx*, *0 ≤ Ci*_*b*_* <1- Δx* and *1 ≥ Ci*_*c*_ *≥ 1- Δx*	SLG fault (B-G)	Tripping
	*i*_*b*_*(n)* and *i*_*b*_*(n - N*_*s*_*)*			
	*i*_*c*_*(n)* and *i*_*c*_*(n - N*_*s*_*)*			
	*i*_*a*_*(n)* and *i*_*a*_*(n - N*_*s*_*)*	*1 ≥ Ci*_*a*_*≥ 1- Δx*, *1 ≥ Ci*_*b*_*≥ 1- Δx* and *0 ≤ Ci*_*c*_ *<1- Δx*	SLG fault (C-G)	Tripping
	*i*_*b*_*(n)* and *i*_*b*_*(n - N*_*s*_*)*			
	*i*_*c*_*(n)* and *i*_*c*_*(n - N*_*s*_*)*			
	*i*_*a*_*(n)* and *i*_*a*_*(n - N*_*s*_*)*	*0 ≤ Ci*_*a*_* <1- Δx*, *0 ≤ Ci*_*b*_* <1- Δx* and *1 ≥ Ci*_*c*_ *≥ 1- Δx*	*Ci*_*a*_ ≈ *Ci*_*b*_ and *Ci*_*ab*_ = *1* DL (A-B) fault	Tripping
	*i*_*b*_*(n)* and *i*_*b*_*(n - N*_*s*_*)*		*Ci*_*a*_ ≠ *Ci*_*b*_ and *Ci*_*ab*_ *≠ 1* DLG (A-B-G) fault	
	*i*_*c*_*(n)* and *i*_*c*_*(n - N*_*s*_*)*			
	*i*_*a*_*(n)* and *i*_*a*_*(n - N*_*s*_*)*	*1 ≥ Ci*_*a*_*≥ 1- Δx*, *0 ≤ Ci*_*b*_* <1- Δx* and *0 ≤ Ci*_*c*_ *<1- Δx*	*Ci*_*b*_ ≈ *Ci*_*c*_ and *Ci*_*bc*_ = *1* DL (B-C) fault	Tripping
	*i*_*b*_*(n)* and *i*_*b*_*(n - N*_*s*_*)*		*Ci*_*b*_ ≠ *Ci*_*c*_ and *Ci*_*bc*_ *≠ 1* DLG (B-C-G) fault	
	*i*_*c*_*(n)* and *i*_*c*_*(n - N*_*s*_*)*			
	*i*_*a*_*(n)* and *i*_*a*_*(n - N*_*s*_*)*	*0 ≤ Ci*_*a*_* <1- Δx*, *1 ≥ Ci*_*b*_*≥ 1- Δx* and *0 ≤ Ci*_*c*_ *<1- Δx*	*Ci*_*a*_ ≈ *Ci*_*c*_ and *Ci*_*ca.*_ = *1* DL (A-C) fault	Tripping
	*i*_*b*_*(n)* and *i*_*b*_*(n - N*_*s*_*)*		*Ci*_*a*_ ≠ *Ci*_*c*_ and *Ci*_*ca.*_ *≠ 1* DLG (A-C-G) fault	
	*i*_*c*_*(n)* and *i*_*c*_*(n - N*_*s*_*)*			
Module (2) for confirming the fault occurrence, and differentiating between the DL and DLG faults using the cross-coherence factors for the current waves	*i*_*a*_*(n)* and *i*_*b*_*(n)*	*0.25 + Δx ≥ Ci*_*ab*_*≥ 0.25- Δx*, *0.25 + Δx ≥ Ci*_*bc*_*≥ 0.25- Δx* and *0.25 + Δx ≥ Ci*_*ca.*_ *≥ 0.25- Δx*	Normal operation	Blocking
	*i*_*b*_*(n)* and *i*_*c*_*(n)*			
	*i*_*c*_*(n)* and *i*_*a*_*(n)*			
	*i*_*a*_*(n)* and *i*_*b*_*(n)*	*0.25 + Δx ˂ Ci*_*ab*_* < 0.25- Δx*, *0.25 + Δx ˂ Ci*_*bc*_* < 0.25- Δx* or *0.25 + Δx ˂ Ci*_*ca.*_ *< 0.25- Δx*	Fault detection	Tripping
	*i*_*b*_*(n)* and *i*_*c*_*(n)*			
	*i*_*c*_*(n)* and *i*_*a*_*(n)*			
	*i*_*a*_*(n)* and *i*_*b*_*(n)*	*Ci*_*ab*_ = *1*	DL (A-B) fault	Tripping
	*i*_*b*_*(n)* and *i*_*c*_*(n)*	*Ci*_*bc*_ = *1*	DL (B-C) fault	
	*i*_*c*_*(n)* and *i*_*a*_*(n)*	*Ci*_*ca.*_ = *1*	DL (C-A) fault	
	*i*_*a*_*(n)* and *i*_*b*_*(n)*	*Ci*_*ab*_ *≠ 1*	DLG (A-B-G) fault	Tripping
	*i*_*b*_*(n)* and *i*_*c*_*(n)*	*Ci*_*bc*_ *≠ 1*	DLG (B-C-G) fault	
	*i*_*c*_*(n)* and *i*_*a*_*(n)*	*Ci*_*ca.*_ *≠ 1*	DLG (C-A-G) fault	

### Square tripping characteristics

**Fig. 2:**
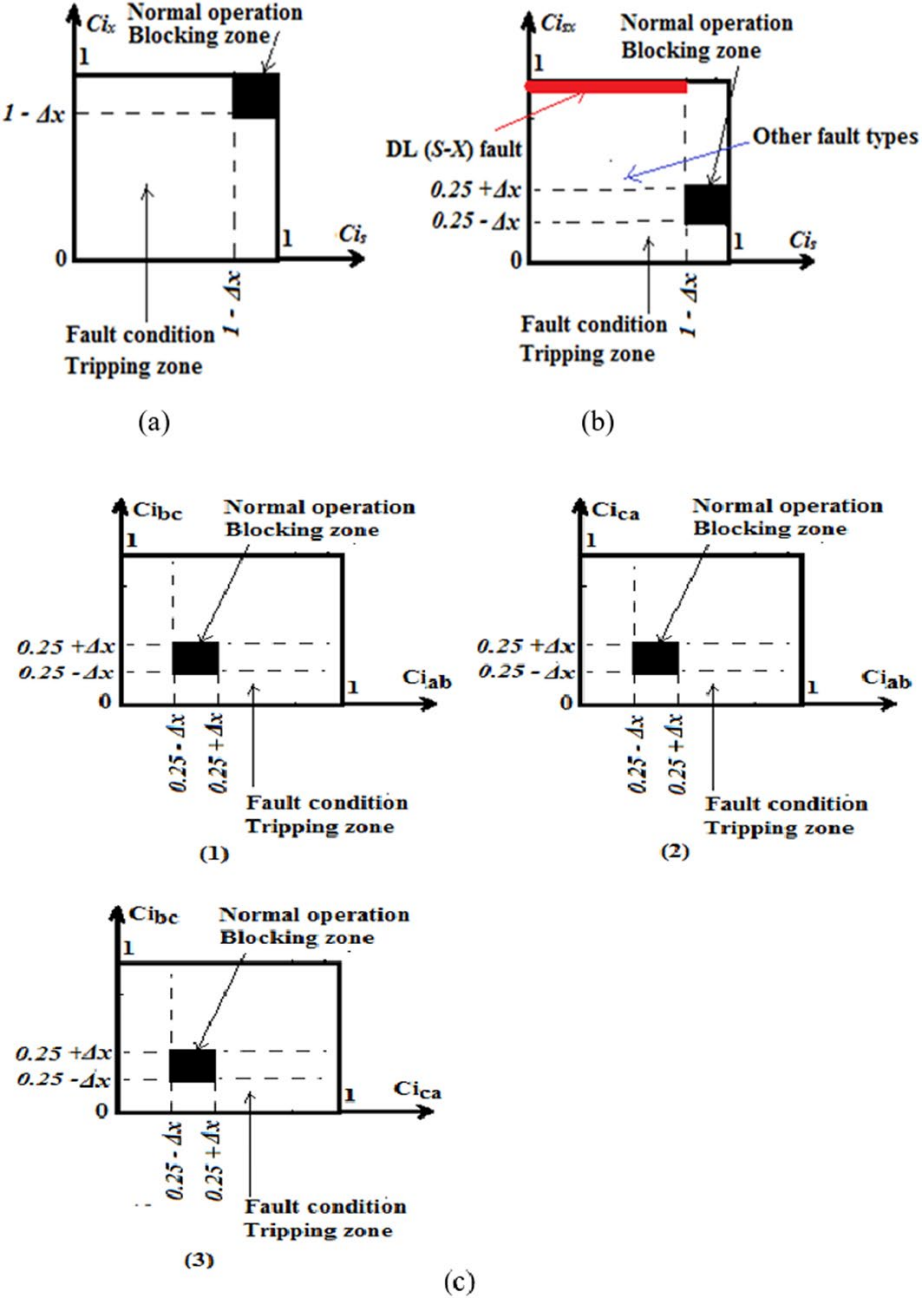
(**a**–**c**) Tripping characteristic curves based on the coherence coefficients. (**a**) Tripping characteristic curve based on auto-coherence coefficients. (**b**) Tripping characteristic curve based on both auto-coherence and cross-coherence coefficients. (**c**) Tripping characteristic curves based on cross-coherence coefficients.

The proposed technique can actually complete its operation and trip TL circuit breaker(s) in the event of fault detection, based on a tripping zone of the coherence characteristic involved. Furthermore, the length of time it takes for the fault locus to traverse the tripping zone. The coherence factors range from 0.0 to + 1.0, as mentioned before. In Fig. 2a–c, new closed-tripping curves with square forms are presented. All tripping curves are restricted and known because they depend on the coherence coefficients. As shown in (Fig. 2a–c), each characteristic has two areas: a tripping area used to isolate TL CBs in the fault situations, and a blocking area used to prevent tripping TL CBs during the normal operating conditions.

Figure 2a depicts the relay operating characteristic curve based on the pair of auto-coherence factors, (*Ci*_*s*_ and *Ci*_*x*_). As illustrated in Fig. 2a, if the operating points of one of the three auto-coherence coefficients fall within at least one tripping zone of the auto-coherence-based characteristic curves, then a fault situation is present. From Fig. 2a, the proposed characteristic curve is able to carry out the following tasks: (1) monitoring and detecting the fault conditions, (2) selecting the faulty phase(s), and (3) classifying the shunt fault type.

Figure 2b shows the relay operating characteristic curve based on the pair of coherence factors, (*Ci*_*s*_ and *Ci*_*sx*_). As demonstrated in Fig. 2b, the power transmission system is subject to both fault and unbalanced currents when the operating points of both auto-coherence and cross-coherence coefficients are inside the tripping zone of this curve. From Fig. 2b, the proposed characteristic curve has the ability to perform the following roles: (1) confirming the presence of the power quality disturbances (PQDs), and (2) differentiating between DL and DLG faults.

Figure 2c presents the relay operating characteristic curves based on the three pairs of cross-coherence factors: (*Ci*_*ab*_ and *Ci*_*bc*_), (*Ci*_*bc*_ and *Ci*_*ca.*_), and (*Ci*_*ca.*_ and *Ci*_*ab*_). As shown in Fig. 2c, if the operating points of one of the three cross-coherence coefficients are situated within at least one tripping zone of the cross-coherence-based characteristic curves, then a current imbalance instance is figured out. From Fig. 2c, the proposed characteristic curves can execute the following functions: (1) assuring the abnormal conditions, and (2) finding and evaluating the three-phase current unbalance.

The TL CBs receives a tripping signal once the protection scheme senses the fault/imbalance situation. If all six coherence coefficients are present within the blocking zones of the relay characteristics, there is no fault nor unbalanced currents condition. As a consequence, the algorithm tripping signal will be active if the fault/imbalance instance is present. Otherwise, it is inactive.

## Power model under test

**Fig. 3:**
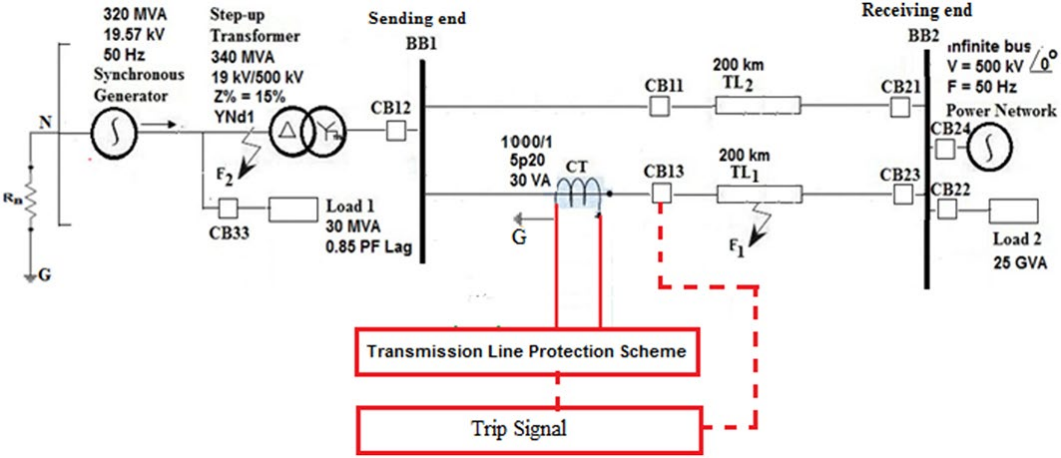
One line diagram of the power system model.

To study a power system model under different operating and fault conditions, the ATP platform is used. In Fig. 3, a single line diagram of the system is shown. The system consists of one synchronous generator (SG) with a step-up transformer (SUT) linked to busbar 1 (BB_1_) and two transmission lines (TL_1_ and TL_2_) that are connected to busbar 2 (BB_2_) of a unified power network. Additionally, there are load 1 linked at the SG terminals, load 2 located at busbar 2 (BB_2_), and eight circuit breakers (CBs) distributed, as shown in (Fig. 3). BB_1_ is the TL_1_ sending end, and BB_2_ is the TL_1_ receiving end. Appendix 1^60^ contains the real data of parameters for the different system components. This study will concentrate on the first transmission line (TL_1_) in order to test the protection based on the coherence function. Therefore, three-phase currents should be measured using protection current transformers installed at the TL_1_ sending end.

## Results analysis

To investigate the accuracy and reliability of the algorithm, the power network under test is simulated using the ATP platform, and signals processing and analysis are employed in MATLAB/M-file environment. The current waveforms, which are measured at the TL_1_ sending end, are used in a discrete form for signal processing and numerical analysis. The sampling frequency of the signals is 5 kHz, which yields 100 samples per cycle of the signal. In this study, the full simulation time is 0.2 s. (i.e., the total number of samples, *N*_*sim*_ = 1000), the fault inception instant (*t*_*i*_) is 0.05 s. (i.e., the fault occurs at the sample order of 250), the fault clearing instant (*t*_*c*_) is 0.2 s, and the shunt faults are existing at 50% of TL_1_ long. To validate the technique performance, the system and fault conditions are changed. This will be accomplished by utilizing different scenarios such as fault type, fault location, fault resistance, fault inception angle and power flow angle. In this work, it is supposed that the electrical power network is loaded before the fault occurrence, and that the pre-fault operating conditions of the simulated power system are tabulated in (Table 2). In Table 3, the fault conditions for each case are given.

Below, there are a few assumptions that are taken into consideration in order to improve the algorithm performance.

1. The same type of three-phase current transformers installed to protect the TL,
2. The same CTRs for all current transformers in the three-phase circuit, and
3. All secondary currents are transformed without core saturation for at least a quarter-cycle starting from the fault inception.

This paper demonstrates only five case studies. The pre-fault results of the proposed scheme are involved in (Table 4).

**Table 2:** Pre-fault operating conditions of the simulated power system.

Electrical component (operating condition)	Data
Operating peak phase voltage (*V*_*1Max*_) of synchronous generator	16.063 kV
Operating peak phase voltage (*V*_*2Max*_) of electrical power network	408.24 kV
F_1op_ of synchronous generator	50 Hz
F_2op_ of electrical power network	50 Hz
Synchronous generator operating power angle (δ_1_)	10^o^
Electrical power network operating power angle (δ_2_)	0^o^
Generator grounding impedance	0.77 Ω
Electrical load 1	10.85 + j 6.72 Ω
Electrical load 2	8.5 + j 5.26 Ω
CB_11_, CB_12_, CB_13_, CB_21_, CB_22_ CB_23_, CB_24_ and CB_33_ Status	Close

**Table 3:** Fault conditions of the case studies.

Case number	Fault type	Fault location	fault inception time, t_f_ (in Sec)	Fault Resistance, *R*_f_ (in Ω)	Current transformer ratio (CTR)	CT burden, *R*_b_ (in Ω)
Case 1	Series fault	*F*_*1*_ (located at 50% of *TL*_*1*_)	0.05	Not applicable	1000/1	0.5 + j0.0
Case 2	SLG shunt fault )*A-G*) through *R*_*f*_ = 500 Ω			500		
Case 3	DLG shunt fault )*A-B-G*)			0.0		
Case 4	DL fault (*A-B*) fault			0.0		
Case 5	3LG shunt fault )*A-B-C-G*)			0.0		

**Table 4:** Pre-fault results of the proposed protection.

Parameter	I_a_(Amp)	I_b_(Amp)	I_c_(Amp)
Peak phase primary value	117	117	117
Peak phase secondary value	0.1165	0.1165	0.1165
Auto-coherence factor	***Ci***_***a***_	***Ci***_***b***_	***Ci***_***c***_
	+ 1.0	+ 1.0	+ 1.0
Cross-coherence factor	***Ci***_***ab***_	***Ci***_***bc***_	***Ci***_***ca.***_
	+ 0.25	+ 0.25	+ 0.25

### Case 1: series fault

**Fig. 4:**
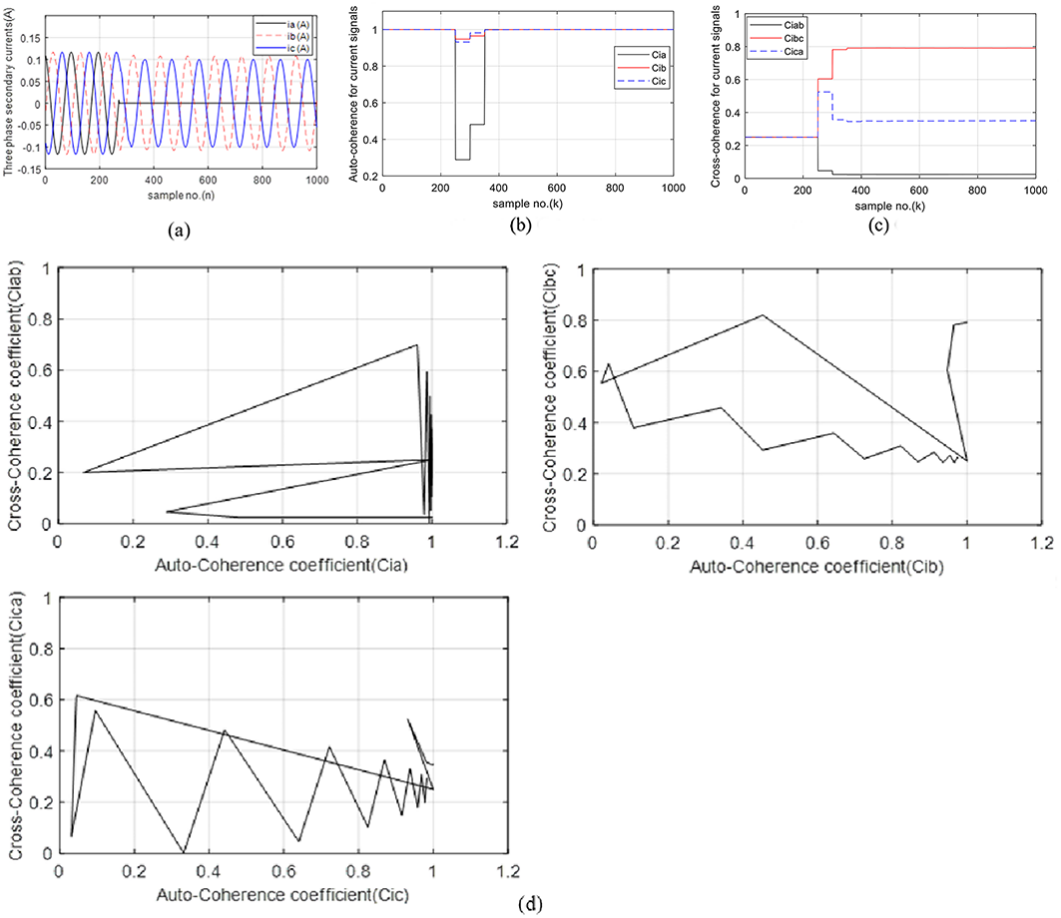
(**a**–**d**) Simulation results for case 1. (**a**) Three-phase secondary currents taken at the TL_1_ sending end. (**b**) Coherence factors (*Ci*_*a*_, *Ci*_*b*_ and *Ci*_*c*_). (**c**) Coherence factors (*Ci*_*ab*_, *Ci*_*bc*_ and *Ci*_*ca.*_). (**d**) Tripping characteristics based on the coherence factors.

In this case, there is a series fault due to a single pole open condition of the three-phase circuit breaker (*CB*_*13*_). In the case of an open “*A*” pole for *CB*_*13*_, unbalanced currents flow in the three-phase system. The series fault initiates at a time (*t*_*i*_) of 0.05 s. from the beginning of the simulation time. Figure 4a illustrates three-phase current waves in the event of the series fault. The figure reveals that the magnitudes of the three-phase current before the fault are normal and balanced for the three-phase system. During the series fault time, the current values of the open “*A*” phase are zero, while the currents of the two healthy phases (“*B*” and “*C*”) are unbalanced, and their amplitudes are lower than the pre-fault current. Figure 4b presents the three auto-coherence factors (*Ci*_*a*_, *Ci*_*b*_ and *Ci*_*c*_) estimated for the three-phase current waves. It is observed that their values are stable, equal, and close to unity (i.e., *Ci*_*a*_ = *Ci*_*b*_ = *Ci*_*c*_ = + 1.0) before the series fault presence. But the three coefficients are uneven and unstable during the first two data windows of the fault time, as shown in (Fig. 4b). During the first two data sets, it is obvious that the values of *Ci*_*b*_ ≈ *Ci*_*c*_ ≈ 0.95, while the *Ci*_*a*_ values are less than + 0.3. This indicates that there is a fault on the phase “*A*” of TL_1_. Three cross-coherence factors (*Ci*_*ab*_, *Ci*_*bc*_ and *Ci*_*ca.*_) are shown in (Fig. 4c). In this case, it is noticed that their values are balanced and close to + 0.25 before the event of series fault, as shown in (Fig. 4c), while their post-fault values become unsymmetrical and unequal. Three tripping characteristics with square shapes are illustrated in Fig. 4d for case 1. Each characteristic consists of one auto-coherence factor and another cross-coherence factor. In this case, it can be evident that the operating points are located within the tripping zone of each characteristic. It is therefore possible to conclude that the values of the six coherence factors are capable of detecting the series fault event, and selecting the faulty phase.

### Case 2: SLG shunt fault with Rf = 500 Ω

**Fig. 5:**
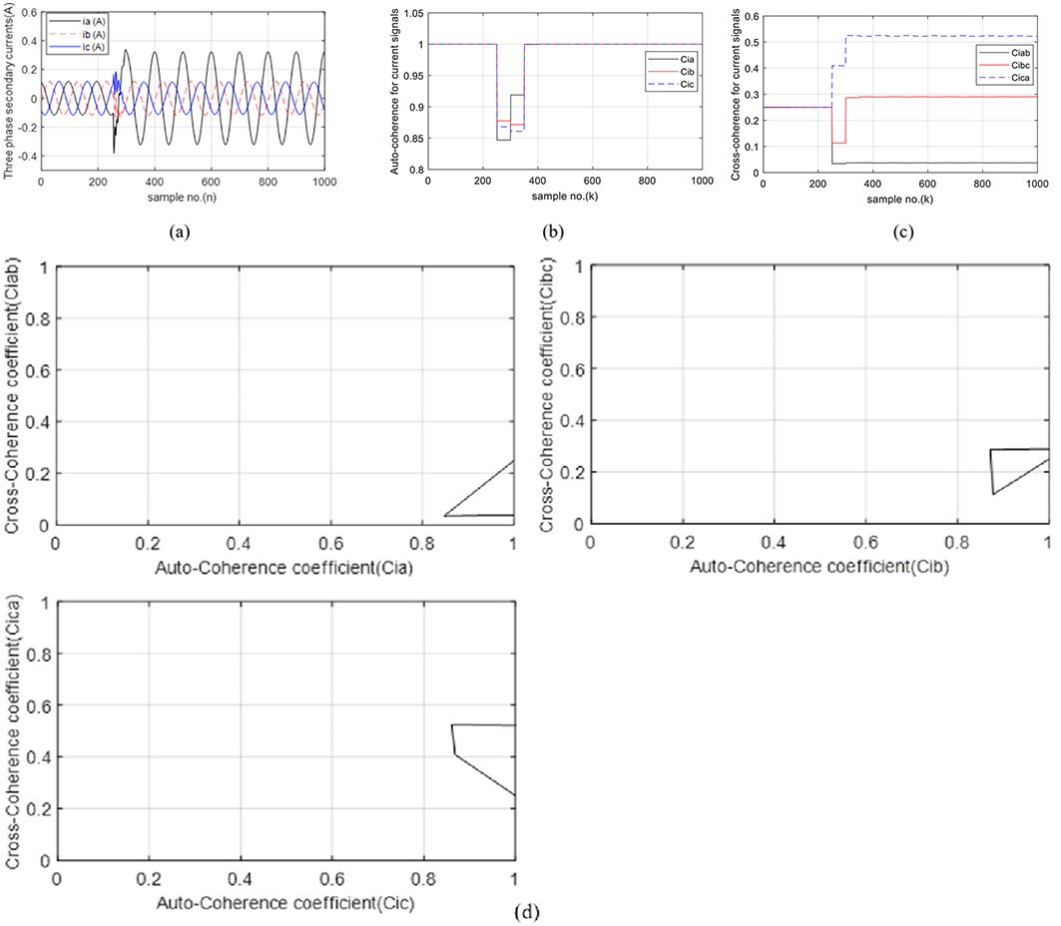
(**a**–**d**) Simulation results for case 2. (**a**) Three-phase secondary currents taken at the TL_1_ sending end. (**b**) Coherence factors (*Ci*_*a*_, *Ci*_*b*_ and *Ci*_*c*_). (**c**) Coherence factors (*Ci*_*ab*_, *Ci*_*bc*_ and *Ci*_*ca.*_). (**d**) Tripping characteristics based on the coherence factors.

The shunt fault is located at the midpoint of TL_1_ in this case. The fault type is *SLG (A-G)* with a fault resistance (*R*_*f*_) of 500 Ω, and the fault starts at a time (*t*_*i*_) of 0.05 s. The curves of three-phase secondary currents taken at the TL_1_ sending end for case 2 are presented in (Fig. 5a). In the figure, the magnitudes of the pre-fault secondary currents are almost constant. The *SLG* fault increases the “*A*” phase current, which is approximately 2.75 times higher than the current before the fault. Figure 5b depicts the three auto-coherence factors (*Ci*_*a*_, *Ci*_*b*_ and *Ci*_*c*_) computed for the three-phase currents (*i*_*a*_, *i*_*b*_ and *i*_*c*_), respectively. In this figure, it is clear that the three factors (*Ci*_*a*_, *Ci*_*b*_ and *Ci*_*c*_) are equal and close to unity before the fault happens, while *Ci*_*a*_≈ 0.84, *Ci*_*b*_ ≈ 0.87 and *Ci*_*c*_≈ 0.86 during the first two data windows of the fault time, as shown in (Fig. 5b). This denotes the fault occurrence on “*A*” phase of TL_1_ because *Ci*_*a*_ is lower than the coherence setting of 1.0 – *Δx =* 0.85. Figure 5c illustrates the three cross-coherence factors (*Ci*_*ab*_, *Ci*_*bc*_, and *Ci*_*ca.*_). It is seen that their values are fixed and close to + 0.25 before the fault presence, while their values are asymmetrical, unequal and outside the domain of 0.25 ± *Δx* during the fault time, as depicted in (Fig. 5c). Figure 5d offers the three tripping characteristics with quadratic forms for case 2. One auto-coherence factor and another cross-coherence factor are used to determine each characteristic. It is noted that the operating points of the three coherence factors (*Ci*_*a*_, *Ci*_*ab*_, and *Ci*_*ca.*_) are placed within the tripping zones of the developed characteristics. This is due to the fault location on the “*A*” phase of the *TL*_*1*_. This event shows that the suggested technique is sensitive because of its ability to recognize the short-circuit current state through the high fault resistance of 500 Ω. Besides, it can choose the faulty phase that is the “*A*” phase of the *TL*_*1*_, and classify the fault type that is *SLG* (*A-G*).

### Case 3: DLG shunt fault with Rf = 0.0 Ω

**Fig. 6:**
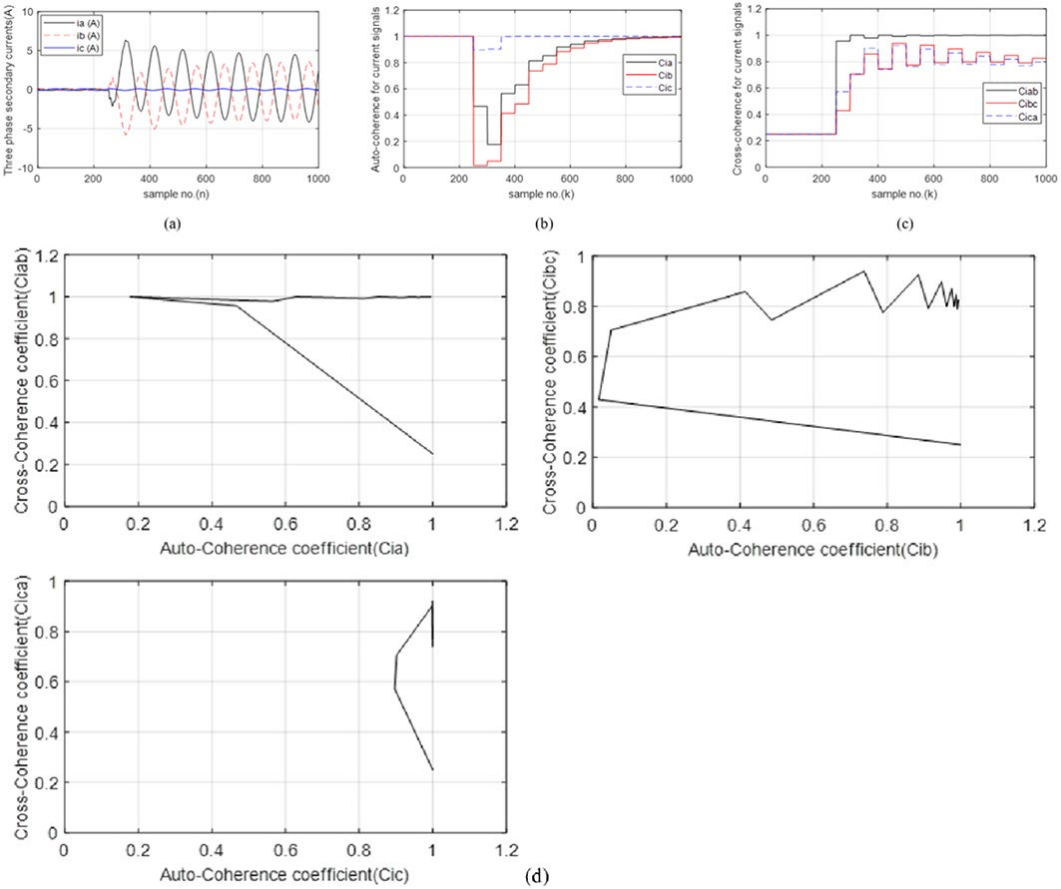
(**a**–**d**) Simulation results for case 3. (**a**) Three-phase secondary currents taken at the TL_1_ sending end. (**b**) Coherence factors (*Ci*_*a*_, *Ci*_*b*_ and *Ci*_*c*_). (**c**) Coherence factors (*Ci*_*ab*_, *Ci*_*bc*_ and *Ci*_*ca.*_). (**d**) Tripping characteristics based on the coherence factors.

In this case, the fault type is changed to bolted *DLG* fault (*A-B-G*). The three-phase currents measured at the *TL*_*1*_ sending end are shown in (Fig. 6a). The two faulty phases (*A* and *B*) have current magnitudes that are nearly 40 times larger than the pre-fault current, while the current value of the healthy phase (*C*) is nearly the same as the pre-fault current. Figure 6b depicts the three auto-coherence factors (*Ci*_*a*_, *Ci*_*b*_, and *Ci*_*c*_). Their values are equilibrium and equal to almost unity before the fault starts. When starting the fault, the values of *Ci*_*a*_ and *Ci*_*b*_ are lower than 0.2, while the values of *Ci*_*c*_ are greater than 0.85. These values indicate that the *A* and *B* phases are faulty, but the *C* phase is healthy. Figure 6c plots the three cross-coherence factors (*Ci*_*ab*_, *Ci*_*bc*_, and *Ci*_*ca.*_). Before the fault inception, their values are identical and equal to nearly + 0.25, while their values are out of the range restricted from 0.1 to 0.4 during the fault period. Therefore, this confirms that the *TL*_*1*_ state is defective. Figure 6d depicts the three tripping characteristics for case 3. It is evident that the values of the five coherence factors (*Ci*_*a*_, *Ci*_*b*_, *Ci*_*ab*_, *Ci*_*bc*_, and *Ci*_*ca.*_) are located within the tripping zones of the developed characteristics. This means that the fault classification is *DLG*, and it is present in the ‘*A* and *B*’ phases of the TL_1_. It is obvious from the obtained results, the three auto-coherence factors (*Ci*_*a*_, *Ci*_*b*_, and *Ci*_*c*_) can define the *DLG* fault. Furthermore, the three cross-coherence factors (*Ci*_*ab*_, *Ci*_*bc*_, and *Ci*_*ca.*_) can be used to assure the fault occurrence, and the fault type is *DLG*. It is not a DL fault since the post-fault values of the factor *Ci*_*ab*_ are below 0.8 (i.e., the factor *Ci*_*ab*_ computed between the two faulty phases is not equal to unity). It is observed from the results, the proposed algorithm (based on the six coherence factors calculated for the current waves) is capable of finding out the fault situation, discriminating the faulty phases that are “*A*” and “*B*” phases of *TL*_*1*_, and classifying the fault type that is *DLG* (*A-B-G*).

### Case 4: DL fault with Rf = 0.0 Ω

**Fig. 7:**
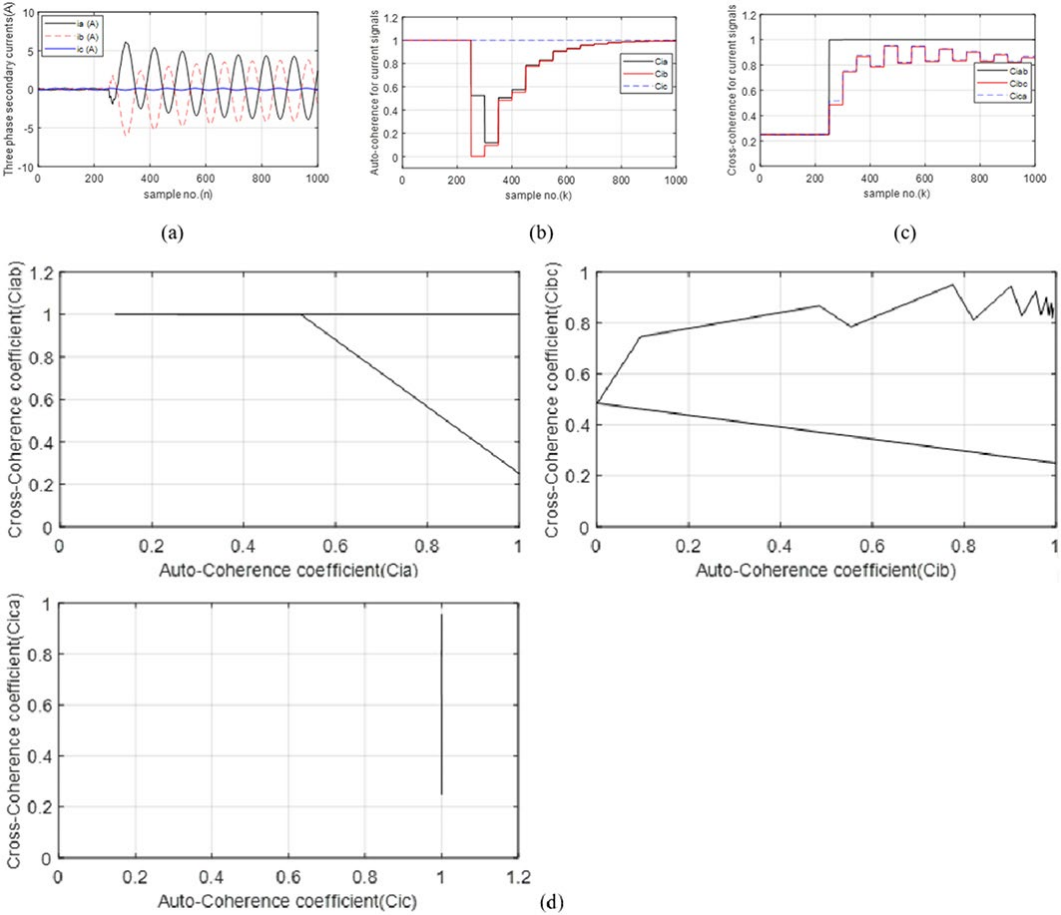
(**a**–**d**) Simulation results for case 4. (**a**) Three-phase secondary currents taken at the TL_1_ sending end. (**b**) Coherence factors (*Ci*_*a*_, *Ci*_*b*_ and *Ci*_*c*_). (**c**) Coherence factors (*Ci*_*ab*_, *Ci*_*bc*_ and *Ci*_*ca.*_). (**d**) Tripping characteristics based on the coherence factors.

The fault type in this case is a bolted *DL* fault (*A-B*) free of grounding. Figure 7a presents the curves for the three-phase currents transformed at the *TL*_*1*_ sending end. During the fault period, the current magnitudes of the two faulty phases are identical, but their directions are different. The post-fault currents of the two faulty phases (*A* and *B*) are nearly 38 times the pre-fault current, while the current magnitude of the healthy phase (*C*) is nearly the same as the pre-fault current. Figure 7b introduces the three auto-coherence coefficients (*Ci*_*a*_, *Ci*_*b*_ and *Ci*_*c*_). Before the fault inception, their values are stable, equal and close to unity. When the fault occurs, the values of *Ci*_*a*_ and *Ci*_*b*_ are less than 0.15, while the values of *Ci*_*c*_ are close to unity. Thus, these values affirm that the *A* and *B* phases are faulty, but the *C* phase is healthy. Figure 7c illustrates the three cross-coherence coefficients (*Ci*_*ab*_, *Ci*_*bc*_, and *Ci*_*ca.*_). Their pre-fault values are symmetrical and equal to about + 0.25. In this case, it is observed that *Ci*_*a*_ ≈ *Ci*_*b*_, *Ci*_*c*_ ≈ +1.0, *Ci*_*bc*_ ≈ *Ci*_*ca.*_, *Ci*_*ab*_ ≈ +1.0 and *i*_*a*_*(n)*_*= −−*_*i*_*b*_*(n)* during the fault time. From the results, it is obvious that the present algorithm can find the fault condition, choose the faulty phases of TL_1_ that are *A* and *B*, and classify the fault kind that is a *DL* (*A-B*) fault free of grounding. In this study, the technique can determine the fault type whether *DLG* or *DL* by calculating the cross-coherence factor computed between the two faulty phases (*A* and *B*), where if *Ci*_*ab*_ ≈ +1.0, then the fault type is *DL*. Otherwise, it is *DLG*. Figure 7d plots the three tripping characteristics based on the six coherence factors of the current waveforms for case 4. It is noticed that the values of the five coherence factors (*Ci*_*a*_, *Ci*_*b*_, *Ci*_*ab*_, *Ci*_*bc*_, and *Ci*_*ca.*_) are located within the tripping zones of the developed characteristics.

The simulation results reveal that the proposed technique is accurate, secure, dependable and reliable to determine the faulted phases, and distinguish between DL isolated and grounded faults without adding any extra algorithm to carry out this task. In addition, the algorithm is fast because the selected data set is within one cycle.

### Case 5: 3LG fault with Rf = 0.0 Ω

**Fig. 8:**
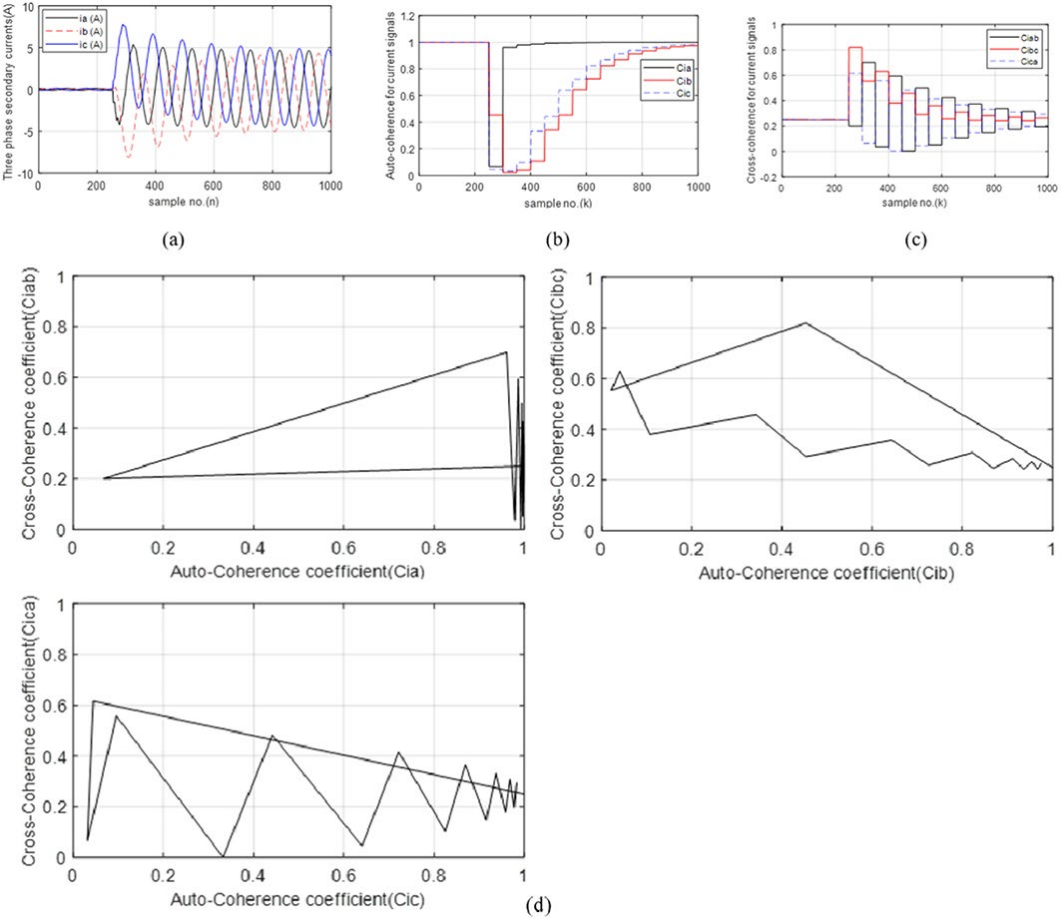
(**a**–**d**) Simulation results for case 5. (**a**) Three-phase secondary currents taken at the TL_1_ sending end. (**b**) Coherence factors (*Ci*_*a*_, *Ci*_*b*_ and *Ci*_*c*_). (**c**) Coherence factors (*Ci*_*ab*_, *Ci*_*bc*_ and *Ci*_*ca.*_). (**d**) Tripping characteristics based on the coherence factors.

In the present case, the fault type is a bolted *3LG* fault (*A-B-C-G*). Figure 8a presents the three-phase currents transformed at the *TL*_*1*_ sending end. In this case, it is noticed that the three-phase currents are higher than the pre-fault currents; the post-fault currents are nearly 40.5 times the pre-fault current. As depicted in (Fig. 8b), the pre-fault values of the three factors (*Ci*_*a*_, *Ci*_*b*_, and *Ci*_*c*_) are balanced, similar and close to + 1.0. But, these factors are less than + 0.5 during the first data set of the fault time. Figure 8c shows the three cross-coherence factors (*Ci*_*ab*_, *Ci*_*bc*_, and *Ci*_*ca.*_). Their pre-fault values are symmetrical and equal to roughly + 0.25, while their post-fault values are out of the scope bounded between 0.1 and 0.4. Therefore, the *TL*_*1*_ situation is abnormal. Figure 8d depicts the three tripping characteristics with quadruple forms for case 5. It is shown that the values of the six coherence factors (*Ci*_*a*_, *Ci*_*b*_, *Ci*_*c*_, *Ci*_*ab*_, *Ci*_*bc*_, and *Ci*_*ca.*_) are settled inside the tripping areas of the proposed characteristics. As a result, the fault classification is *3LG* (*A-B-C-G*).

From the results of the extensive simulation cases, the suggested scheme is smart, effective, and quick for detecting the situations of the series and ten shunt faults, selecting the faulty phase(s), and classifying the fault type. This demonstrates that the coherence function is considered a proper fault detector, classifier, and discriminator of faulty phase(s) for power TL.

The quantified findings are presented in Table 5 for the first two data windows of the fault time.

**Table 5:** Post-fault results of the proposed protection scheme.

Case number	Fault type	The obtained results during the first two windows of the fault time					
		Auto-coherence			Cross-coherence		
		Ci_a_	Ci_b_	Ci_c_	Ci_ab_	Ci_bc_	Ci_ca_
Pre-fault		1.0	1.0	1.0	0.25	0.25	0.25
Case 1	Series fault (open A phase)	*Ci*_*a*_ < 0.3	*Ci*_*b*_ ≈ *Ci*_*c*_ ≈ 0.95	*Ci*_*b*_ ≈ *Ci*_*c*_ ≈ 0.95	Out of the range	Out of the range	Inside the range
Case 2	SLG shunt fault )*A-G*) through *R*_*f*_ = 500 Ω	*Ci*_*a*_ < 0.84	*Ci*_*b*_ ≈ 0.87	*Ci*_*c*_≈ 0.86	Out of the range	Inside the range	Out of the range
Case 3	DLG shunt fault )*A-B-G*)	*Ci*_*a*_ < 0.2	*Ci*_*b*_ < 0.2	*Ci*_*c*_ > 0.85	Out of the range *Ci*_*ab*_ ≠ + 1.0	Out of the range	Out of the range
Case 4	DL fault (*A-B*) fault	*Ci*_*a*_ ≈ *Ci*_*b*_ < 0.5	*Ci*_*a*_ ≈ *Ci*_*b*_ < 0.5	*Ci*_*c*_ ≈ *1.0*	Out of the range *Ci*_*ab*_ ≈ +1.0	Out of the range *Ci*_*bc*_ ≈ *Ci*_*ca.*_,	Out of the range *Ci*_*bc*_ ≈ *Ci*_*ca.*_,
Case 5	3LG shunt fault )*A-B-C-G*)	*Ci*_*a*_ < 0.1	*Ci*_*b*_ < 0.5	*Ci*_*c*_ < 0.1	Out of the range	Out of the range	Out of the range

The selected *Δx* is 0.15, the setting of the auto-coherence factors is 1.0 − *Δx*, and the setting of the cross-coherence factors is 0.25 ± *Δx.*

The coherence algorithm has been examined in situations involving noise in the current signals, stable power swings, unstable power swings, CT saturation, and currents through high fault resistances. In addition, cases in which the fault occurs at sending and receiving ends of the transmission line. Table 6 summarizes the results of other case studies under the effect of these situations for the simulated power system. As presented in Appendix (2), case studies for stable and unstable power swings have been discriminated using the coherence algorithm. As illustrated in Table 6, the algorithm is blocked in the instance of stable power swings, while it responds in the event of unstable power swings. This is one of the advantages of the proposed algorithm based on the coherence statistic.

**Table 6:** The relay responses for other situations.

Ser. no.	Case type	coherence algorithm action (blocking/tripping)	Correct/incorrect action
1	Stable power swings	Blocking	Correct action
2	Unstable power swings	Tripping	Correct action
3	Noise in the current signals	Blocking (The reasonable coherence settings and the data window size can be used to avoid the effect of the acceptable harmonics content in the current signals.)	Correct action
4	CT saturation	The same author presented another paper^57^ that explains the methodology for CT saturation detection using the coherence algorithm. In^57^, case studies were described for busbar protection with CT saturation extent.	Correct action
5	Weak infeed and outfeed (in which the fault currents are reduced and resemble load currents)	Tripping (To make the algorithm more sensitive for fault detection, the size of the data window and coherence setting deviations should be reduced, which can be selected a sub-cycle period.)	Correct action
6	Faults located at sending and receiving ends of the transmission line	Tripping The proposed algorithm installed at the sending end can monitor and protect the entire transmission line. In other sense, the proposed algorithm constructed at the sending end can monitor and detect the faults at the receiving end of the power system under test. Hints: The relay action depends on the impedance specification of the transmission line required to be protected and the fault resistance. Additionally, the developed algorithm placed at the receiving end can monitor and protect the same transmission line.	Correct action

## Salient features of the proposed technique

### Advanced technique features

The present technique has several distinctive characteristics as follows:

1. The coherence function is able to detect different fault events, select the faulty phase(s), and classify all ten types of shunt faults. Furthermore, it can recognize series faults,
2. It is highly sensitive because fault events through large fault resistances can be identified,
3. It is capable of distinguishing between DL and DLG faults without any extra algorithm,
4. This approach has a quick response, since the operating time taken by this approach is about half-cycle,
5. Its security, sensitivity, and response speed can be set by adjusting the data set amount or the coherence-setting margin,
6. Three-phase current measurements are sufficient for the proposed scheme to be processed,
7. New tripping curves are proposed, which have square shapes and are restricted and determined because they are based on the coherence factors,
8. The performance of the technique is independent on the specification of the current transformers and power system components,
9. The coherence method works like a digital low-pass filter to reduce the effect of DC contents and transient harmonics on measured currents,
10. The technique can be implemented in practice,
11. It is advantageous with light data transmission burden because it only requires three-phase current measurements at the TL sending or receiving end,
12. Data synchronization systems are not required, and
13. It is called high-reliability because it is immune to issues like fault time, fault inception angle, fault location, fault resistance or fault type.

### Comparison

Table 7 illustrates a comparison between the proposed protection technique based on the coherence criterion and other recently published techniques used to protect transmission line systems.

**Table 7:** A comparison between the proposed and published protection techniques.

Ser. no.	Item	The proposed protection technique	The published protection techniques
1	Required measurements	The three-phase currents measured at one end of the transmission line are required for estimating the six coherence coefficients. This results in swift data transfer and processing.	Several existing methods require three-phase voltages and currents at one or two ends of the transmission line^1,4,6,7,13,34,37,38^. Thus, these existing methods have a greater computational burden than the proposal.
2	Main basic	It is contingent on the mean-squared coherence (MSC) estimators, which are applied to only three-phase currents.	Numerous methods are employed to estimate the impedance required for processing the protection of the transmission lines^4,6,49^.
3	Its function	It has an online function.	Certain protection methods have an offline function.
4	Sampling size	The sampling rate of the developed method is 100 samples per cycle.	In^5,26,32,36,39^, the techniques used a higher sampling rate to protect the transmission line. The sampling rate exceeded 100 samples per cycle, leading to a delay in the relay operation^5,26,32,36,39^.
5	Data window/RMS concept	In the developed algorithm, the coherence coefficients are computed using the moving data set concept. The data set quantity can be modified in accordance with the protection characteristics required and the prevailing conditions of the power network.	The RMS values of voltage and current measurements are used to compute the impedance needed to operate the existing impedance relays^4,6,7,13,37,49^. Other techniques used the data set concept, which could be modified in line with the protection characteristics required and the prevailing operating conditions of the power system^12^.
		The approach significantly outperforms other RMS quantities-based techniques that require several cycles to compute the relay operating time. This fast response reduces electrical and mechanical stresses during fault conditions, providing improved protection for power transmission systems and ensuring power network stability.	
6	Tripping characteristic curves	The tripping and blocking zones within the proposed quadratic characteristic curves derived from the coherence coefficients can be used to distinguish between a transmission line fault and none, respectively. These curves are restricted from 0.0 to + 1.0.	The characteristic curves of the impedance relays had different shapes depending on the impedance estimation These curves were not limited because of the variation in the parameters of different transmission system sizes^4,6,30,31,49,53^.
			The tripping characteristics curves of the impedance relays depend on the parameters of the power transmission lines and the specification of the voltage and current transformers^4,6,30,31,49,53^.
7	Relay settings	The numerical values of the data set size and the specified coherence deviations can be used to alter the requirements of the protection attributes, such as speed, reliability, and sensitivity.	The settings of the impedance relays should be neatly set to avoid any protection malfunction^4,30,31,53^.
		There is no need to estimate the coherence settings for changing the operating and restraining regions of the coherence characteristic curves, and it is unnecessary to modify the relay settings in response to changes in transmission system topologies/dimensions.	Numerous conventional relays of transmission lines protection need extensive offline studies to prepare their settings^1^.
			In^3,7,8,10,11,13,]^^14,18,27^, a variety of mathematical models were applied. As a result, the algorithms needed diverse setting values to be set.
8	Algorithm complexity degree	It is easy to use in practice due to its simple mathematical formulas.	Several techniques are significantly complicated, making them impractical^18,19,22,36,41^.
9	Multiple functions	Many protection functions can be performed for transmission systems using the coherence coefficients quantified for the three-phase currents as follows:	In several protection techniques, only one or two protection functions were implemented^13,31^.
		(1) Detection of the series and shunt faults,	
		(2) Determination of the severity of shunt faults,	
		(3) Classification of TL shunt faults,	
		(4) Discrimination between DL and DLG faults,	
		(5) Differentiation between symmetrical and unsymmetrical faults,	
		(6) Identification of three-phase current imbalance, and	
		(7) Assessment of three-phase current imbalance.	
10	Digital filter	The data window concept, which is used to estimate the coherence coefficients, serves as a low-pass filter.	Some protection systems may need additional low-pass filters to remove or alleviate ripples in voltage or current waves^28,29^.
11	Response to stable power swing	The methodology does not operate during stable power swings.	Some protection strategies were incorrectly employed during stable power swings^4,6,30,31,49^.
			The variations in the topology and parameters of the power transmission system affect the operation of several protection strategies^1,28^.
12	Redundancy protection	The utilization of auto-coherence and cross-coherence algorithms increases protection reliability and redundancy.	Numerous conventional methods lack the attributes of protection reliability and redundancy^4,13,23,31^.
13	Protection integration	The present algorithm is capable of integrating with other conventional protection techniques to improve the protection reliability.	The absence of protection integration in numerous techniques resulted in a reduction in the protection reliability^18,19^.
14	Protection speed	The protection system is contingent upon the direct coherence models calculated for the transmission line current measurements. As a consequence, the operating speed of the coherence-based technique is superior to that of the alienation-based technique.	In^8,11^, the approaches were dependent on the alienation estimator derived from the coherence/correlation estimator obtained for the transmission line current measurements. Hence, the operational speed of the alienation-based model is lower than that of the coherence-based model.
			In^7,13,37^, the methods used Phasor Measurement Units (PMUs) that made a significant delay in fault detection.
15	Protection precision	The simulation findings reveal that the suggested scheme has the ability to accurately identify fault events.	The protection accuracy of several impedance relays is low owing to the elevated fault resistance, which reduces the RMS magnitude of the fault current^13,17^.
16	Protection reliability	The proposed approach achieves the requirements of the protection reliability. The failure of the relay operation indicates the unreliability of the protection system.	Many fault detection methods are unreliable in the instance of symmetrical faults^4–6,32^. Other existing protective relays achieve the requirements of the protection reliability.
17	Protection security	Increasing both the size of the data window and the coherence setting deviations can enhance the protection security of the proposed technique. The increase in the coherence setting deviations increases the blocking region and decreases the tripping region, with each other, inside the setup of characteristic curves.	The protection security of the conventional impedance relays can be enhanced by reducing the impedance curve and increasing the relay operating time^4,6,30,31^.
18	Protection dependability	Reducing both the amount of the data window and the coherence setting deviations can improve the protection dependability of the proposed technique. The reduction in the coherence setting deviations decreases the blocking region and increases the tripping region, with each other, inside the established characteristic curves.	The protection dependability of the conventional impedance relays can be improved by increasing the impedance curve and reducing the relay operating time^4,6,30,31^.
19	protection sensitivity	The protection sensitivity of the proposed technique can be boosted by reducing both the data set size and the coherence setting deviations of the suggested tripping characteristic curves based on the coherence coefficients. The coherence setting deviations are beneficial for modifying both blocking and tripping regions placed in the proposed characteristic curves. Therefore, these settings are capable of adjusting the protection sensitivity.	High threshold impedance can enhance the protection sensitivity of the conventional impedance relays^1,4,6,30,31^.
		The proposed quadratic curves are limited because they are based on the coherence coefficients that expand from 0.0 to + 1.0. Thus, the reduction in the blocking region results in an increase in the tripping region within the proposed curves.	The size of the impedance tripping curve relies on the parameters of the transmission lines and the specifications of the voltage and current transformers^1,4,6,30,31^.
		The entire area of the proposed characteristic curve is independent of the parameters of the transmission lines or the specifications of the current transformers.	Only one tripping curve can be selected for the impedance-based protection. Consequently, the area of tripping region of the relay characteristic curve is fixed^4,6,30,31,49,53^.
20	Application fields	Different scales of transmission lines can be protected with the coherence-based protection system.	It could be applied to protect diverse sizes of transmission lines against different fault scenarios^40–53^.
		In addition, the scheme can be applied to identify series and shunt faults in both traditional and smart grids with different voltage and power ratings.	Additionally, some existing techniques were applicable in both traditional and smart grids with a variety of voltage standards^26,36,39^.
		It can be applied to protect many of power system components, such as transmission lines, AC generators^54,56,59^, AC motors, power transformers and busbars^57^.	Conventional impedance relays are used to protect only power transmission lines^26,36,39^.
21	Experimental verifications	The investigation of the coherence approach involves thorough computer simulations^54^ and experimental verifications^59^.	The impedance approaches were verified through comprehensive simulation programs and practical experiments^40–53^.

## Conclusions

A numerical analysis of the coherence function for detecting, selecting phase(s), and classifying TL faults has been presented in this paper. The method has used six coherence factors that are calculated only for the current measurements taken at the TL sending end. In order to evaluate the technique’s performance, numerous simulation studies have been conducted on a segment of a power grid with real data parameters. The system model has been simulated under different operating and fault conditions, such as fault type, fault location, fault resistance, fault inception angle and power flow angle. The simulation results have confirmed the high capabilities of the proposed protection for detection, classification, and faulty phase(s) identification of various faults. In addition, it is simple, smart, exact, robust, stable, and reliable, and it is not affected by the specification of the TL parameters or current transformers. Moreover, its response is fast as the relay operating time is within a half-cycle time, and it is sensitive because the faults through high fault resistance are detectable. Furthermore, the relay response speed, sensitivity, and security levels can be set by adjusting the data set amount and/or the coherence setting margin. In conclusion, a new method has been introduced that uses square forms of tripping curves based on the coherence values (which are similar to the per-unit values). Each curve includes two restricted areas: tripping and restraining. The protection can be active within the tripping area for the abnormal states of the system, while it can be inactive within the restraining area for the normal operating states. In the following items, the proposed method based on the coherence function is superior to the existing methods based on the impedance calculation:

1. Both the tripping and restraining zones are restricted because their boundary ranges between 0.0 and 1.0, and the normal operating points are neatly predetermined,
2. The coherence coefficients are similar to the per-unit values,
3. There is no need to calculate the setting parameters and the specification of the transmission lines and instrument transformers are not required,
4. A low-pass filter and a data synchronization system are unnecessary,
5. The algorithm demands only instantaneous measurements of the current signals acquired at one of the two terminals of the transmission line,
6. The same coherence algorithm is able to identify, classify, and discriminate the faulty phases. Besides, it can find out and measure the current unbalance level,
7. No additional algorithm is needed to differentiate between DL and DLG faults, and.
8. The coherence technique can distinguish between stable and unstable power swings, and perform an appropriate decision-making process.

## Electronic Supplementary Material

Below is the link to the electronic supplementary material.

Supplementary Material 1

Supplementary Material 2

## Data Availability

All data generated or analysed during this study are included in this published article [and its supplementary information files].

## Abbreviations

*i*_*a*_*(n)*, *i*_*b*_*(n)* and *i*_*c*_*(n)*: The three-phase current waves at sample *‘n’* measured at the TL sending end of phases *A*, *B* and *C* respectively
*i*_*s*_*(n)*: The current signal for every sample *n* of phase ’*S*’
*i*_*x*_*(n)*: The current signal for every sample *n* of phase ’*X*’
*i*_*s*_*(n-Ns)*: The instantaneous phase current signal *(i*_*s*_*)* at sample one-cycle prior to *n* of ’*S* phase’
*n*: The *n*’th sample (in the time domain)
*S* and *X*: The phase designation *A*,* B* or *C*; but they are not the same phase
*Ci*_*s*_(*k*): The auto-coherence factor, on a given frequency (*k*), calculated between each two successive data windows shifted from each other by one-cycle interval of the current signal *(i*_*s*_*(n)* and *i*_*s*_*(n - N*_*s*_*))* of the *‘S’* phase
*Ci*_*sx*_*(k)*: he cross-coherence factor, on a given frequency (*k*), calculated between the two current waves *(i*_*s*_*(n)* and *i*_*x*_*(n))* for the two phases *‘S’* and *‘X’*, respectively
*DFT*: Discrete fourier transform
*RMS*: The root mean square
*Δx*: The coherence setting deviation; it lies within the range of 0.0 and + 0.25
*K*: The *k*’th frequency component
*N*_*s*_: The number of samples per cycle used in the simulation, (*N*_*s*_ = 100 samples/cycle)
*N*: The number of samples per data window used in the technique (*N ≤ N*_*s*_); it is selected = *N*_*s*_/2
*T*_*c*_: The periodic cycle, (*T*_*c*_ = 20 *mSec*)
*F*_*c*_: The fundamental frequency of one periodic cycle, (*F*_*c*_ = 50 Hz)
*T*_*s*_: The sampling time interval, (*T*_*s*_ = 0.2 *mSec*)
*F*_*s*_: The sampling frequency rate, (*F*_*s*_ = 5 kHz)
*ω*: The angular velocity of the power system (ω = 2* π*f)
*R*_*f*_: The fault resistance imposed from the faulted point on SG stator winding to the ground point in the case of the ground fault, or inserted between the two faulted phases in case of the phase fault.
*t*_*f*_: The fault inception time
*N*_*f*_: The sample index at which the fault occurs
*t*_*c*_: The fault clearing time
*N*_*c*_: The sample index at which the fault clears
*N*_*sim*_: The full number of samples per the simulation time.
*Ө*: The fault inception angle
*α*: The power system angle
*SLGF*: Single line-to-ground fault
*DLGF*: Double line-to-ground fault
*DLF*: Double line fault
*3LGF*: Three line-to-ground fault
*ATP*: Alternative transient program
*SG*: Synchronous generator
*TL*: Transmission line
*R*_*n*_: Generator grounding impedance through the neutral point
*V*_*n*_: The nominal voltage of the power transmission line
*I*_*n*_: The nominal current of the power transmission line
*SLD*: Single line diagram
*PF*: Power factor
*FD*: Fault detection
*FL*: Fault location
*BB*: Busbar
*CB*: Circuit breaker
*CT*: Current transformer
*CTR*: Current transformer ratio
*R*_*b*_: The current transformer burden (it is 1 *Ω*)
*R*_*CT*_: The current transformer secondary winding resistance
*R*_*lead*_: The lead resistance connected between the current transformer terminals and the burden
*V*_*1Max*_: The peak phase voltage of synchronous generator
*V*_*2Max*_: The peak phase voltage of the electrical network
*F*_*1op*_: The operating frequency of synchronous generator
*F*_*2op*_: The operating frequency of the electrical network
*δ*_*1*_: The operating power angle of synchronous generator
*δ*_*2*_: The operating power angle of the electrical network
