# Supplementary material for: Transmission line faults detection and classification using new tripping characteristics based on statistical coherence for current measurements
Source: Sci Rep. 2025 Mar 12;15:8487. doi: 10.1038/s41598-025-87577-5 (PMC11903867; doi:10.1038/s41598-025-87577-5)
Supplement: Supplementary file 1 — Supplementary Material 1 [file 41598_2025_87577_MOESM1_ESM.pdf]

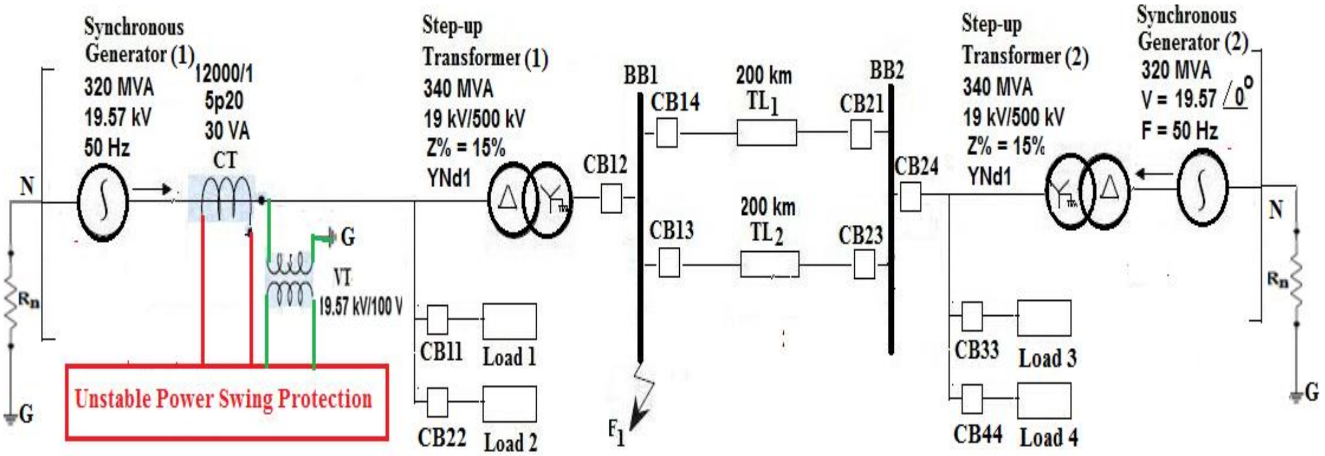

Fig. 3 Single line diagram of the power system model.

Table 4: Specifications of the power system components.

System component specifications	Data
**Synchronous generator 1 (Sending source):**	
Rated Volt-ampere	*320 MVA*
Rated line voltage	*19.57 kV*
voltage phasor angle	20^0
Rated frequency	*50 Hz*
Number of poles	*2*
Neutral grounding impedance (R_n)	*0.77 Ω*
**Synchronous generator 2 (Receiving source):**	
Rated Volt-ampere	*320 MVA*
Rated line voltage	*19.57 kV*
voltage phasor angle	0^0
Rated frequency	*50 Hz*
Number of poles	*2*
Neutral grounding impedance (R_n)	*0.77 Ω*
**Main Transformers (Transformers 1, and 2)::**	
Rated Volt-ampere	*340 MVA*
Transformation voltage ratio	*19.57 kV /500 kV*
Connection primary/secondary	*Delta/Star earthed neutral*
Primary winding impedance (Z_p)	*0.0027 + j0.184 Ω*
Secondary winding impedance (Z_s)	*0.7708 + j 61.8 Ω.*
Vector group	*YNd1*
Z%	*15%*
**Auxiliary Transformers (Transformers 1, and 2)::**	
Rated Volt-ampere	*30 MVA*
Transformation voltage ratio	*19.57 kV/ 6.6 kV/ 6.6 kV*
Connection primary/secondary	*Delta/Star earthed neutral/Star earthed neutral*
Primary winding impedance (Z_p)	*0.029776 + j0.4894 Ω*
Secondary winding impedance (Z_s)	*0.0039 + j 0.0261 Ω.*
Tertiary winding impedance (Zs)	*0.0039 + j 0.0261 Ω.*
Vector group	*YNd1d1*
Z%	*18%*

**Transmission Lines (1&2):**<br>Positive sequence R<br>Zero sequence R<br>Positive sequence XL<br>Zero sequence XL<br>Positive sequence 1/Xc<br>Zero sequence 1/Xc<br>Transmission line long (Km)	 *0.0217 Ω /km*<br>*0.247 Ω /km*<br>*0.302 Ω /km*<br>*0.91 Ω /km*<br>*3.96 μ℧ /km*<br>*2.94 μ℧ /km*<br>*200 Km*
**Electrical Loads (Loads 1, 2, 3, and 4):**<br>Electrical Load	 2.5 + j 1.5 Ω at the sides of *6.6 kV* for the secondary and tertiary windings of the auxiliary transformers
**Current Transformers (CTs):**<br>CTR<br>Rated burden<br>Class	 *12000/1*<br>*30 VA*<br>*5p20*
**Voltage Transformers (VTs)**<br>VTR<br>Rated burden<br>Class	 *11kV/100V*<br>*35 VA*<br>*0.5*

4. Simulation results and analysis

On the ATP platform, measurements are taken at the first SG load terminals, for five cases of power swings. Then the protection algorithm is processed in MATLAB code. The algorithm' performance is verified under various parameters such as load flow levels, load angles, fault types, fault locations and fault time distance. This study will focus exclusively on the variation of the first SG load angles. This work will concentrates on five case studies of the first SG with different operating load angles (δ_1) as follows: 20°, 26.5^o, 27°, 30°, and 40°, respectively. There is a variation in the load flow during the normal operating conditions (i.e., before the 3LG fault occurrence) if the first SG load angle (δ_1) changes from one scenario to another. A Three Line-to-Ground (3LG) fault is located at the fault point named F_1 existing on the first busbar (BB_1), assuming that the fault resistance is null, as shown in Fig. 3. Furthermore, consider that the fault is temporary and that it is automatically cleared. The simulation program generates the voltage and current data at a frequency rate of 5.0 kHz. The full simulation time is 100 cycles long. The fault inception time is 0.402 Second (corresponding to the sample order of 2010), and the fault clearing time is 0.502 Second (corresponding to the sample order of 2510). This means that the fault time distance is 5 cycles long.

The operating and fault conditions of the power grid during the simulation time of the five case studies are noted in Table 5. The simulation results demonstrate that the different operating load angles will affect the SG stability after fault clearing. Moreover, the coherence estimator has the capability to detect the unstable power swings, and assess the transient stability of the power system.

Table 5: Operating and fault conditions of the power grid for the five case studies.

Case number	Fault classification	Operating load angle (δ_1)	Fault location	fault inception time, t_f (in Sec) and fault clearing time, t_c (in Sec)	Fault resistance, R_f (in Ω)	Voltage and Current Transformers Ratios (CTR and VTR)	CT burden, R_b (in Ω)
1	Three line-to-ground (A-B-C-G) fault	20.0^o	F_1 (located at the first busbar, BB_1)	fault inception time (t_f) = 0.402 Sec (at sample order of 2010), and the fault clearing time (t_c)= 0.502 Sec (at sample order of 2510)	0.0	VTR = *11000/100*, and CTR = 12000/1	0.5 + j0.0
2		26.5^o					
3		27.0^o					
4		30.0^o					
5		40.0^o					

4.1 Case 1: Stable power swing (δ_1= 20.0^o)

This case assumes that the first SG operates at a load angle (δ_1) of 20.0^o, and the second SG runs at a load angle (δ_2) of 0.0^o. Figs. 4(a-d) show the electrical waveforms taken at the first SG output for case study 1. Fig. 4(a) depicts the three-phase primary voltage waveforms (v_a, v_b and v_c), Fig. 4(b) presents the three-phase primary current waveforms (i_a, i_b and i_c), Fig. 4(c) illustrates the three-phase primary active power waveforms (P_a, P_b and P_c), and Fig. 4(d) offers the three-phase primary reactive power waveforms (Q_a, Q_b and Q_c) for case study 1.

Figs. 5(a-b) introduce the calculated variables taken at the first SG output for case study 1. Fig. 5(a) presents the total primary active and reactive power waveforms (P and Q). Fig. 5(b) shows the calculated load angle per each phase. Figs. 6(a-b) offer the post-fault operating points of the relay characteristics based on the coherence coefficients computed for the current waves for case study 1. Fig. 6(a) manifests the auto-coherence coefficients (Ci_a, Ci_b, and Ci_c), and Fig. 6(b) offers the cross-coherence coefficients (Ci_{ab}, Ci_{bc}, and Ci_{ca}).

Before the fault presence, it is noticed the following points:

- The electrical waveforms are fixed and balanced for the three phases,
- The total active and reactive powers are approximately constant,
- The computed power factor (PF) angles are stationary,
- The three auto-coherence factors (Ci_a, Ci_b, and $Ci_{c,}$) are stable, normal and equal to nearly +1.0,
- The three cross-coherence factors (Ci_{ab}, Ci_{bc}, and Ci_{ca}) are constant, normal and equal to approximately +0.25,
- The coherence coefficients are stable, normal and nearly equal for the three phases,
- The operating points of the six coherence coefficients are existing in the blocking zone of each proposed characteristic,

During the fault time, it is clear the following points:

- The first SG speed and power angle increase,
- The total active power (MWs) export decreases and the total reactive power (MVARs) export increases, which agrees with the generator transient stability theory,
- The *3LG* fault causes very fast transient in the three-phase currents, active and reactive powers,
- Most coherence coefficients are disturbed and unequal for the three phases

- The operating points of the coherence values are located in the tripping zone of each proposed characteristic,

- The coherence factors fluctuate and their values prove that the fault type is *3LG*,

After clearing the fault, it is obvious the following points:

- The first SG speed and power angle begin to stabilize,

- There are decent fluctuations in the electrical waveforms,

- The three auto-coherence factors (Ci_a, Ci_b, and Ci_c) are stable, normal and close to +1.0,

- The three cross-coherence factors (Ci_{ab}, Ci_{bc}, and Ci_{ca}) are swing and range between +0.2501 and +0.2684,

- The six coherence coefficients are stable, normal, and roughly equal for the three phases,

- The operating points of all coherence coefficients are placed in the blocking zone of each proposed characteristic,

For case 1, the operating conditions lead to the system is rendered as stable after the fault clearance.

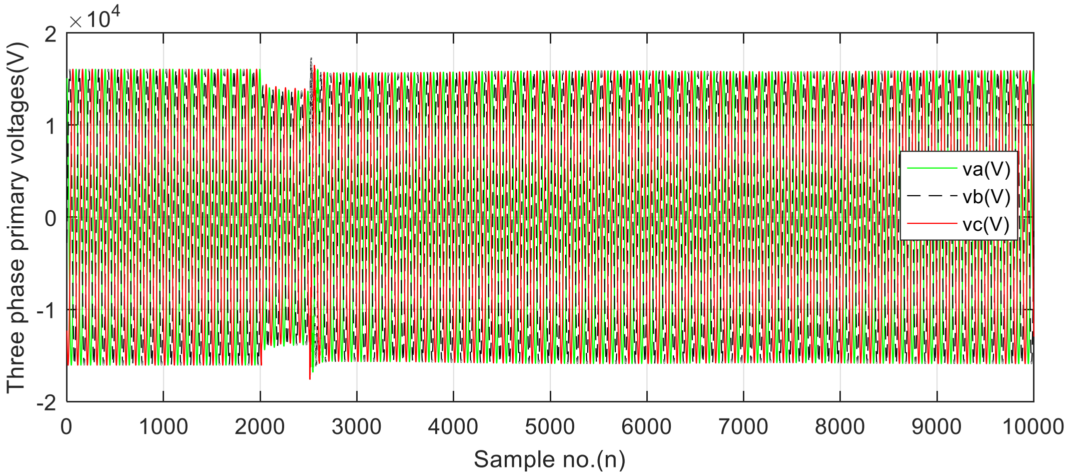

(a)

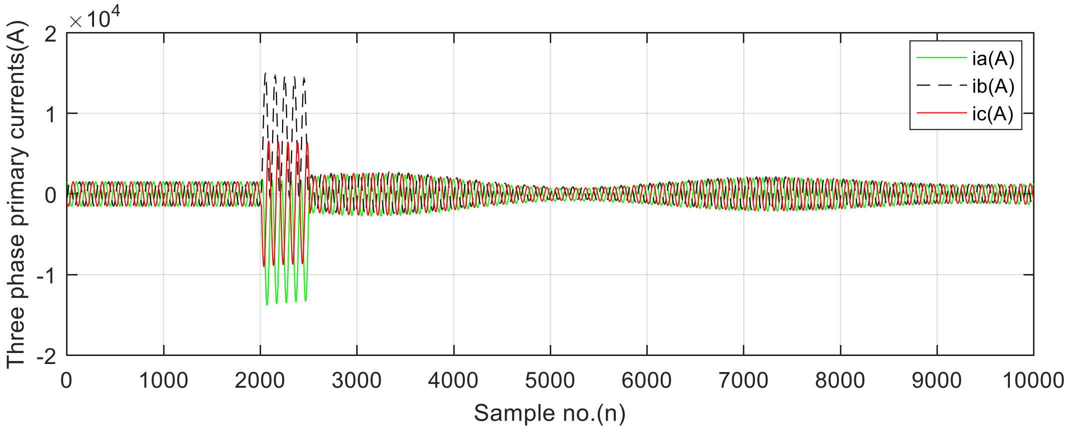

(b)

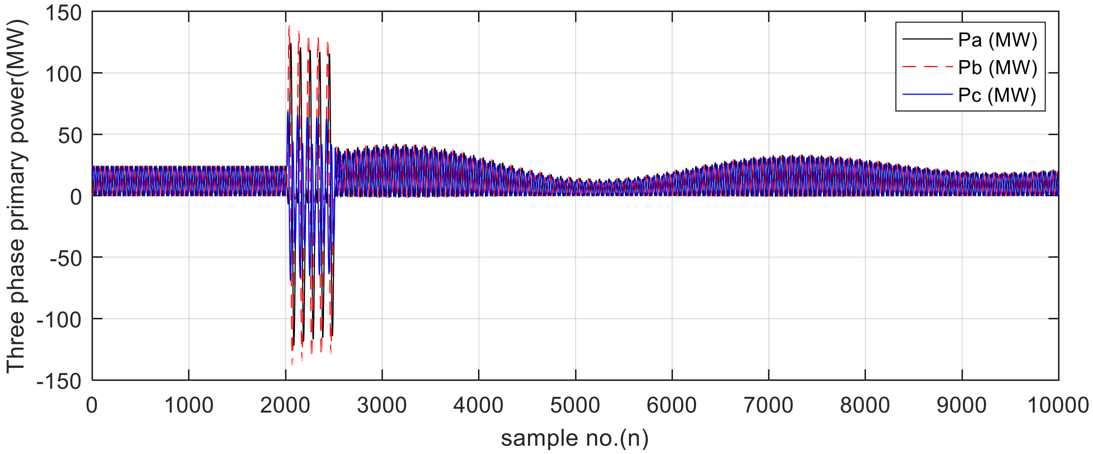

(c)

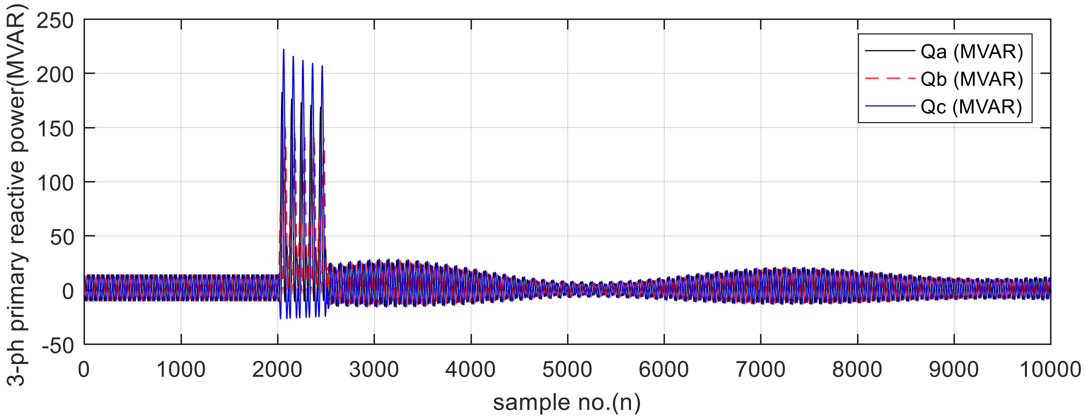

(d)

Figs. 4(a-d) The electrical waveforms taken at the first SG output for case study 1 (Stable power swing). (a) The three-phase primary voltage waveforms (v_a, v_b and v_c), (b) The three-phase primary current waveforms (i_a, i_b and i_c), (c) The three-phase primary active power waveforms (P_a, P_b and P_c), and (d) The three-phase primary reactive power waveforms (Q_a, Q_b and Q_c).

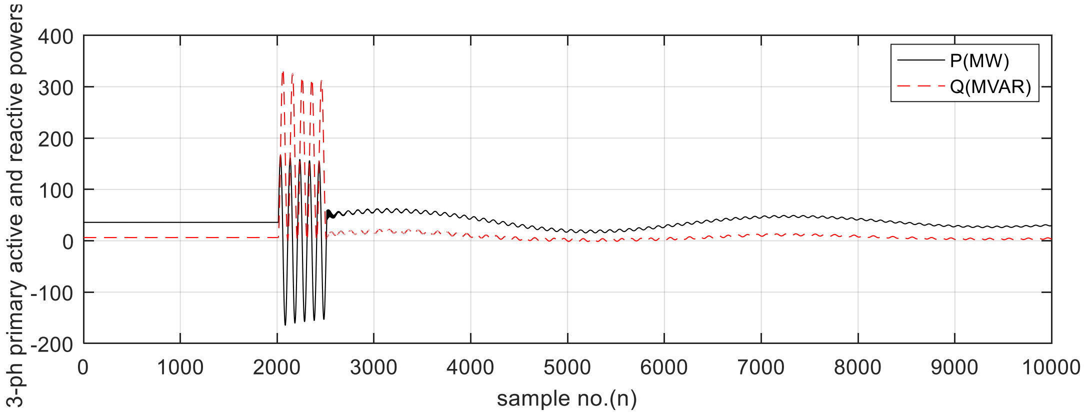

(a)

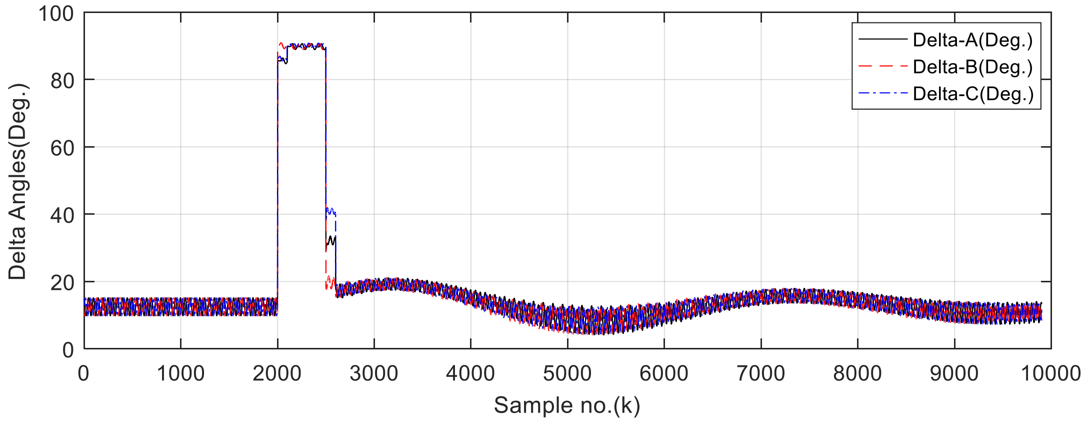

(b)

Figs. 5(a-b) The calculated variables taken at the first SG output for case study 1 (Stable power swing), (a) The total primary active and reactive power waveforms (*P* and *Q*), and (b) The calculated load angle.

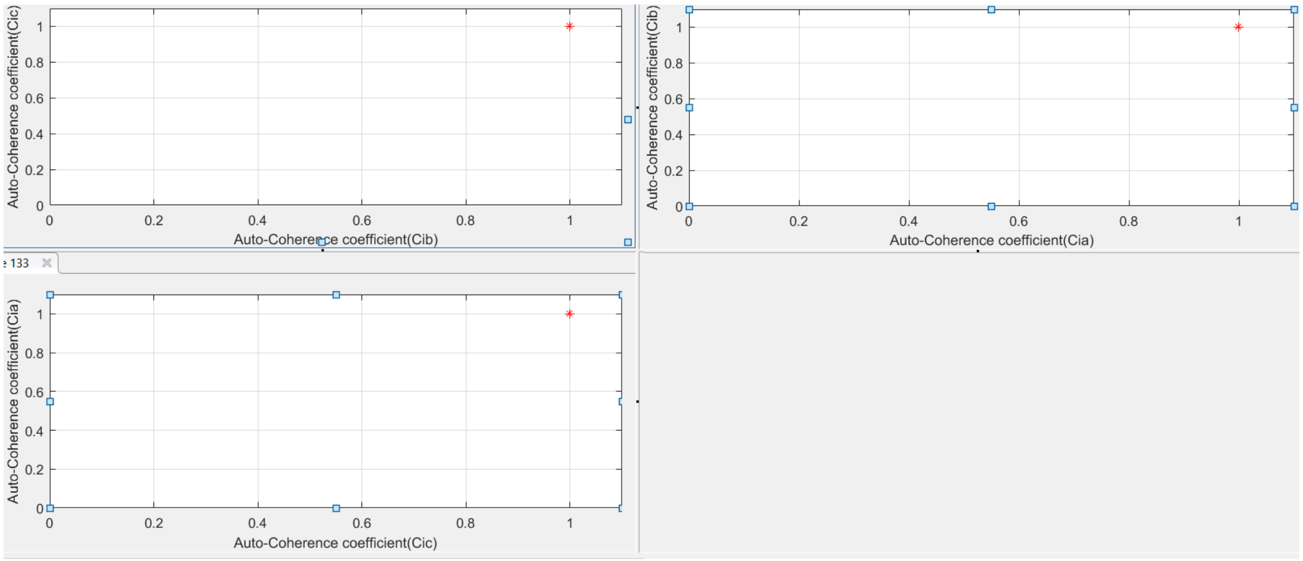

(a)

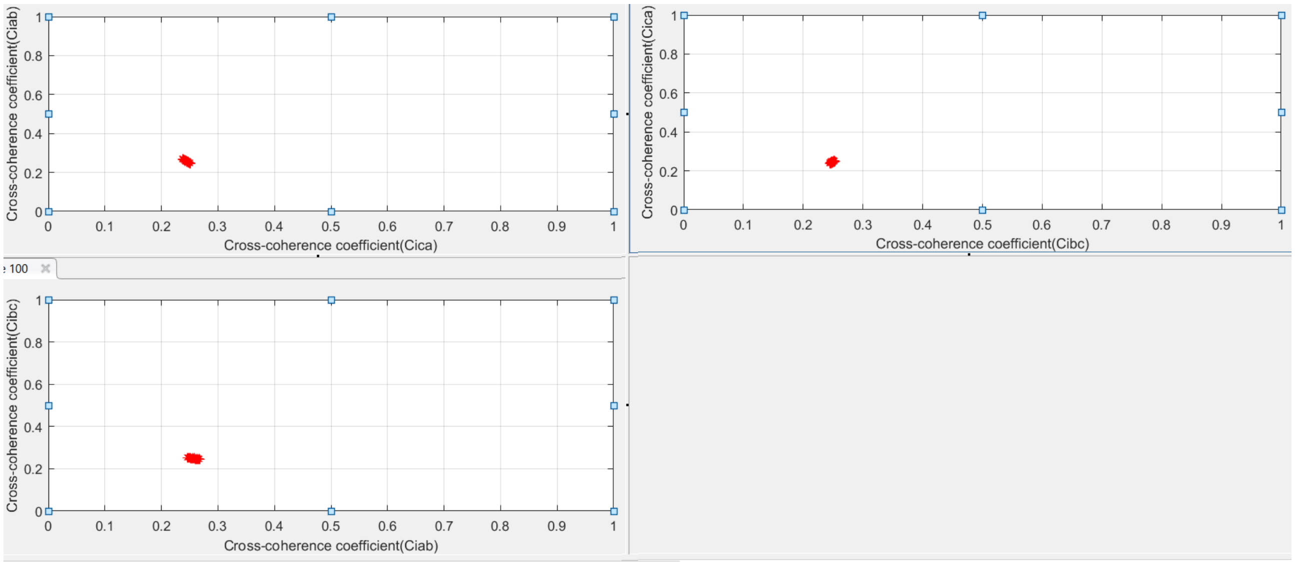

(b)

Figs. 6(a-b) The post-fault operating points of the relay characteristics based on the coherence coefficients computed for the current waves for case study 1 (Stable power swing). (a) the auto-coherence coefficients (Ci_a, Ci_b, and Ci_c), and (b) the cross-coherence coefficients (Ci_{ab}, Ci_{bc}, and Ci_{ca}).

4.2 Case 2: Critical stable power swing (δ_1= 26.5^o)

In this case, the first SG runs at a load angle (δ_1) of 26.5^o. Before and during the fault occurrence, it is evident that the notes in case 2 are similar to those mentioned in case 1. For case study 2, the electrical waveforms taken at the first SG output are depicted in Figs. 9(a-d). The three-phase primary voltage waveforms (v_a, v_b and v_c) are shown in Fig. 9(a), the three-phase primary current waveforms (i_a, i_b and i_c) are depicted in Fig. 9(b), the three-phase primary active power waveforms (P_a, P_b and P_c) are described in Fig. 9(c), and the three-phase primary reactive power waveforms (Q_a, Q_b and Q_c) are presented in Fig. 9(d).

For case study 2, the calculated variables taken at the first SG output are illustrated in Figs. 10(a-b). The total primary active and reactive power waveforms (P and Q) are exhibited in Fig. 10(a). The calculated load angle per each phase is shown Fig. 10(b). Figs. 11(a-b) display the post-fault operating points of the relay characteristics based on the coherence coefficients computed for the current waves for case study 2. Fig. 11(a) manifests the auto-coherence coefficients (Ci_a, Ci_b, and Ci_c), and Fig. 11(b) offers the cross-coherence coefficients (Ci_{ab}, Ci_{bc}, and Ci_{ca}).

After clearing the fault, it is observed the following items:

- The first SG speed and power angle relatively oscillate,
- There are remarkable swings somewhat in the electrical waves,
- The power angles (δ_s) have acceptable fluctuations,
- The three auto-coherence factors (Ci_a, Ci_b, and Ci_c) range between 0.4189 and 1.0 (i.e. they are within the tripping zone),
- The three cross-coherence factors (Ci_{ab}, Ci_{bc}, and Ci_{ca}) are swing and range between +0.1805 and +0.2741 (i.e. they are within the tripping zone),

- In this case, the six coherence factors (Ci_a, Ci_b, Ci_c, Ci_{ab}, Ci_{bc}, and Ci_{ca}) are abnormal and are located within the tripping area of the proposed characteristics. Therefore, the simulation results affirm the system status is considered a critical stable after clearing the fault.

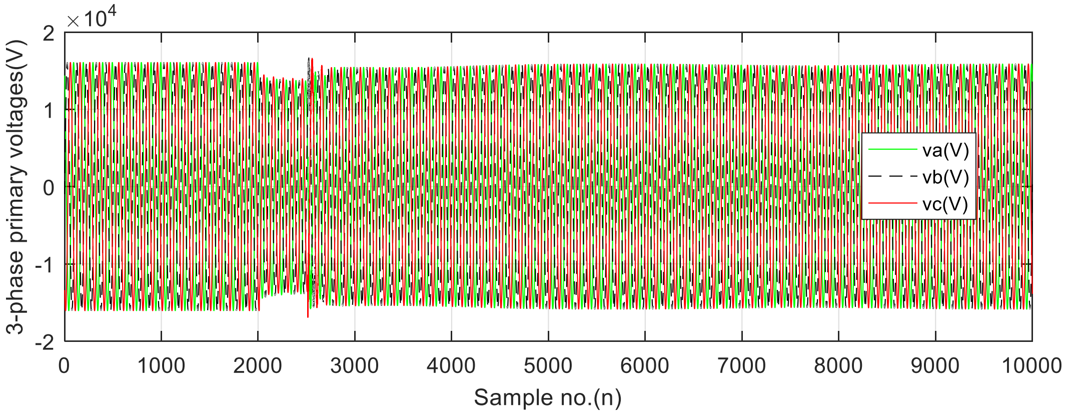

(a)

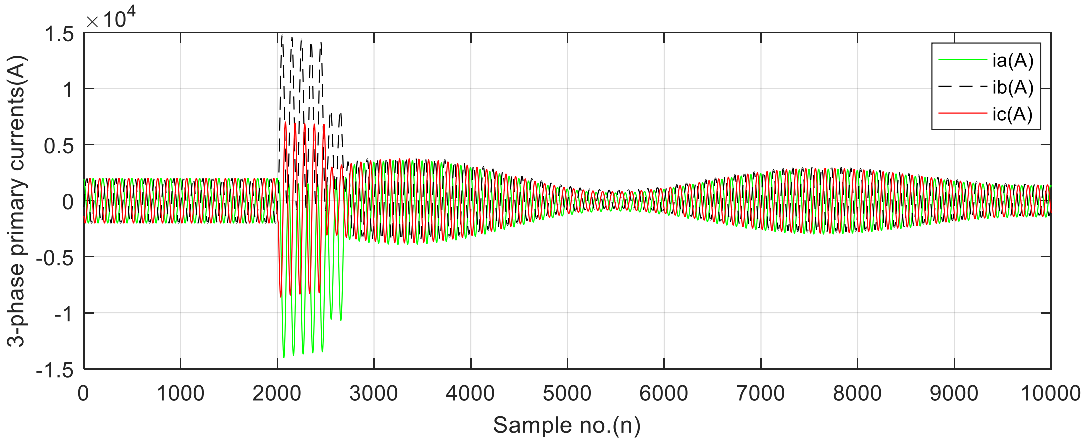

(b)

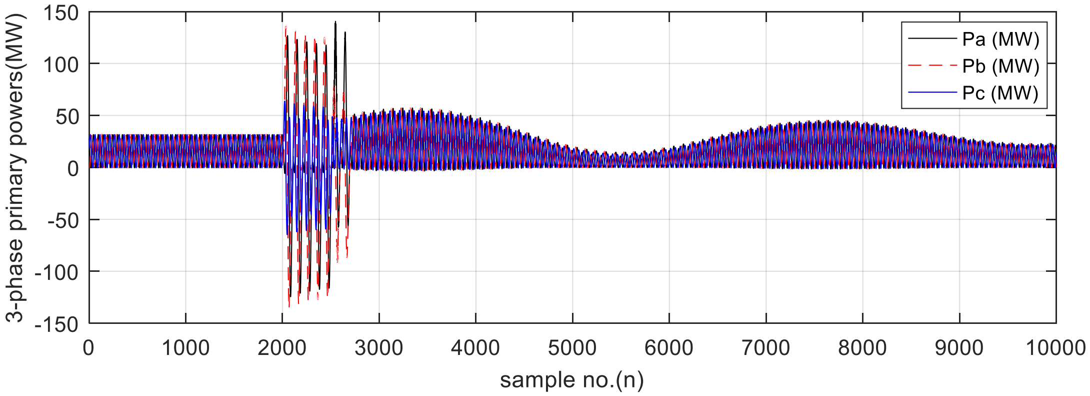

(c)

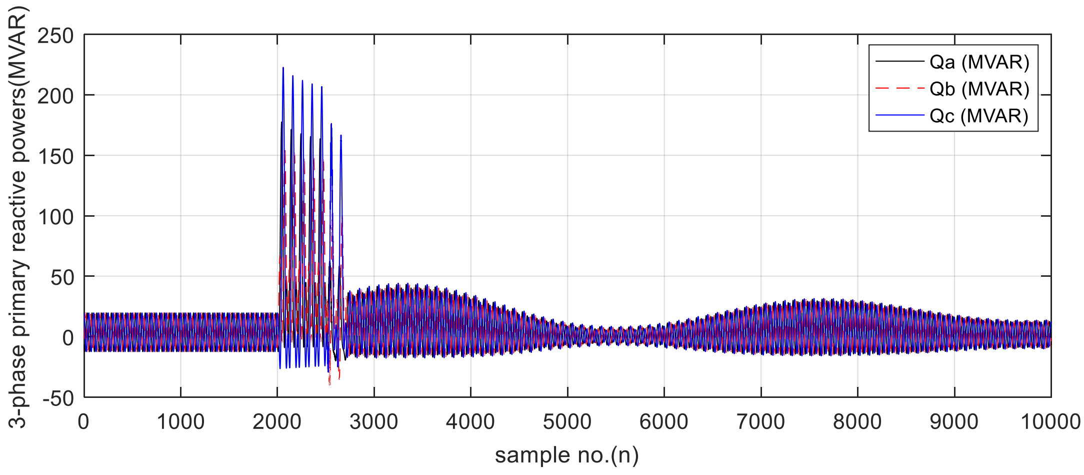

(d)

Figs. 9(a-d) The electrical waveforms taken at the first SG output for case study 2 (Critical stable power swing). (a) The three-phase primary voltage waveforms (v_a, v_b and v_c), (b) The three-phase primary current waveforms (i_a, i_b and i_c), (c) The three-phase primary active power waveforms (P_a, P_b and P_c), and (d) The three-phase primary reactive power waveforms (Q_a, Q_b and Q_c).

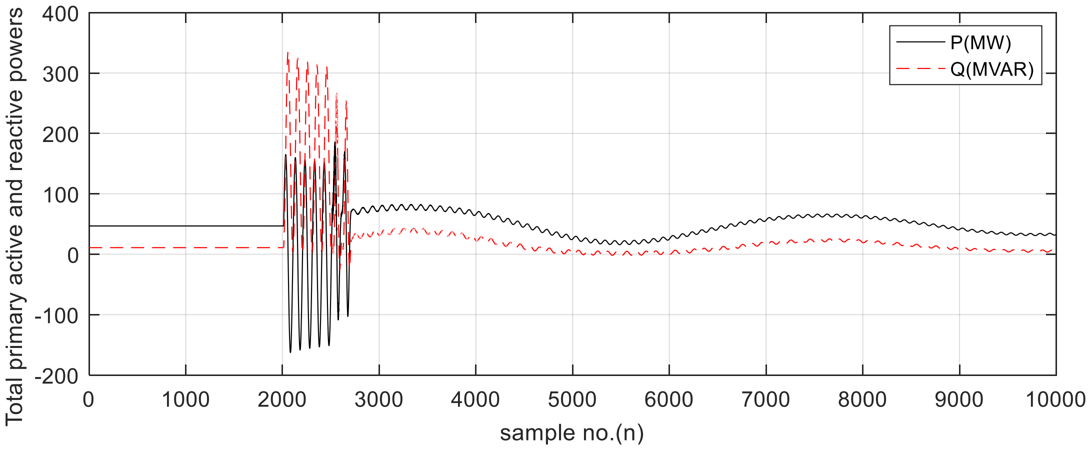

(a)

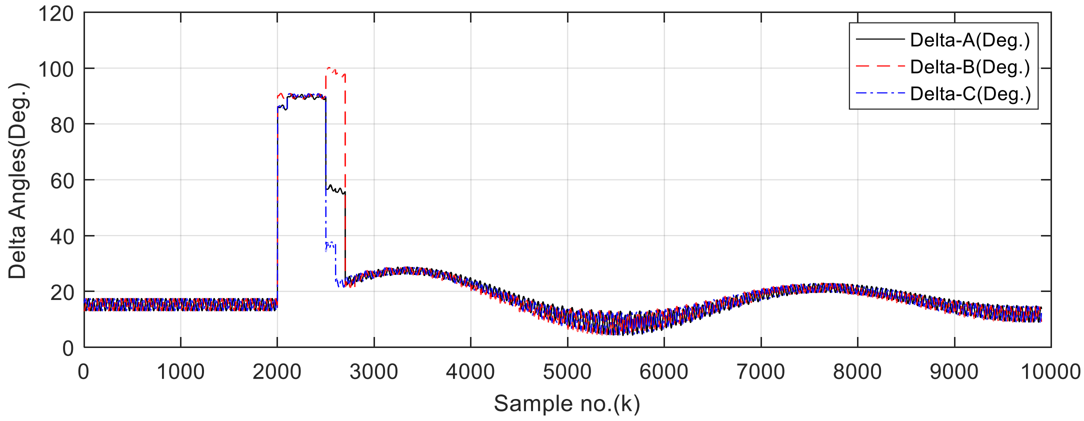

(b)

Figs. 10(a-b) The calculated variables taken at the first SG output for case study 2 (Critical stable power swing), (a) The total primary active and reactive power waveforms (P and Q), and (b) The calculated load angle.

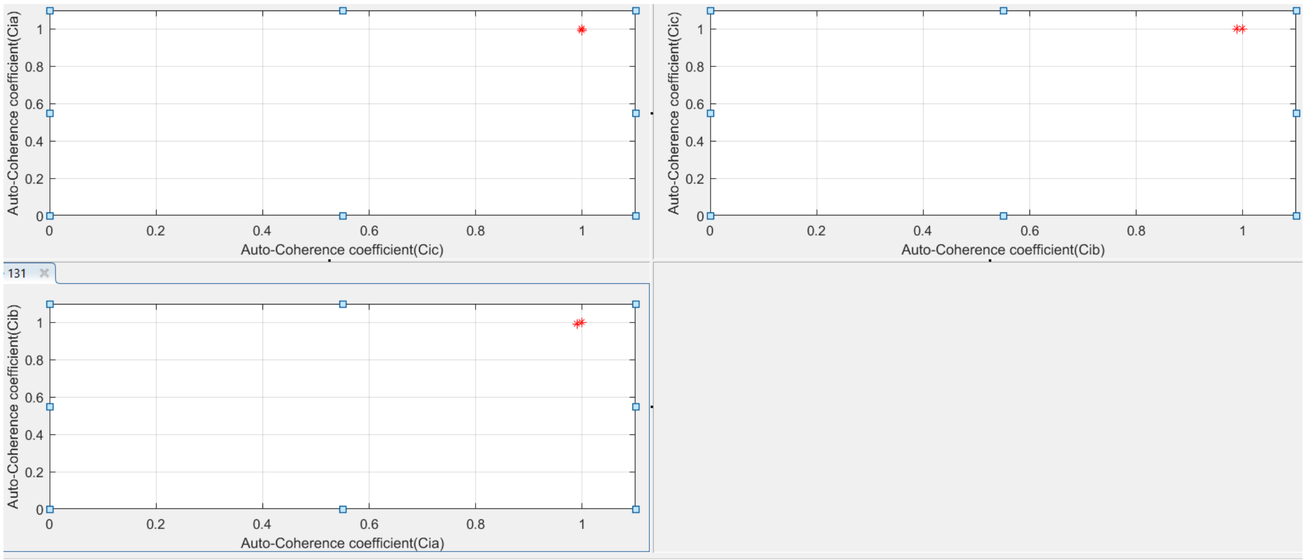

(a)

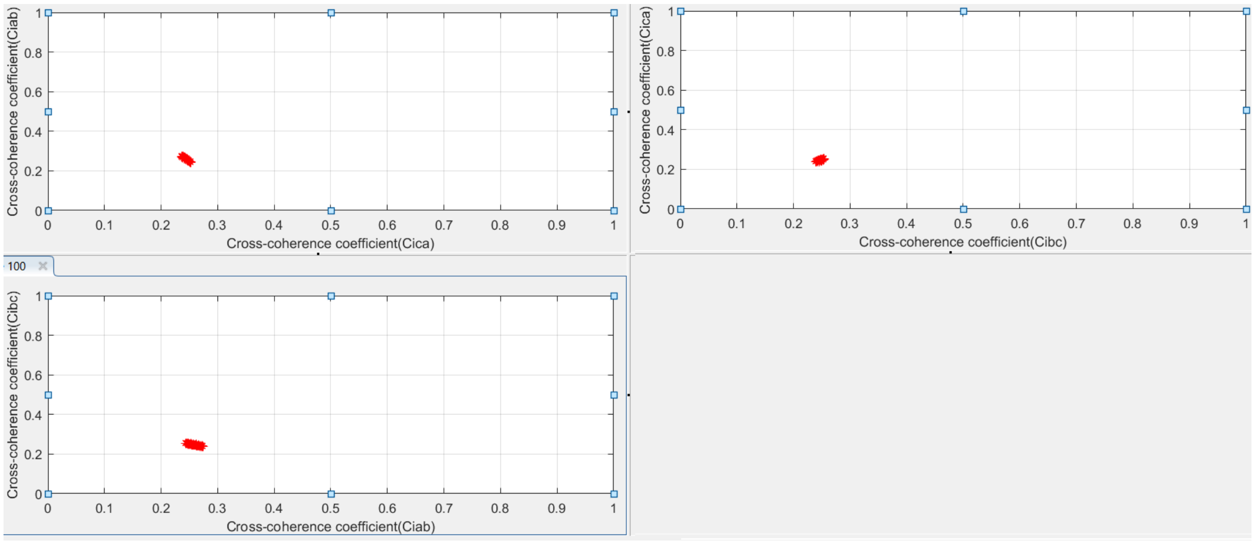

(b)

Figs. 11(a-b) The post-fault operating points of the relay characteristics based on the coherence coefficients computed for the current waves for case study 2 (Critical stable power swing), (a) the auto-coherence coefficients (Ci_a, Ci_b, and Ci_c), and (b) the cross-coherence coefficients (Ci_{ab}, Ci_{bc}, and Ci_{ca}).

4.3 Case 3: Unstable power swing (δ_1= 27.0^o)

In case 3, the first SG operates at a power angle (δ_1) of 27.0^o. Before and during the fault occurrence, it is evident that the notes in case 3 are identical to those mentioned in case 1. Figs. 14(a-d) display the electrical waveforms taken at the first SG output for case study 3. Fig. 14(a) presents the three-phase primary voltage waveforms (v_a, v_b and v_c), Fig. 14(b) exhibits the three-phase primary current waveforms (i_a, i_b and i_c), Fig. 14(c) describes the three-

phase primary active power waveforms (P_a, P_b and P_c), and Fig. 14(d) shows the three-phase primary reactive power waveforms (Q_a, Q_b and Q_c).

Figs. 15(a-b) illustrate the calculated variables taken at the first SG output for case study 3. Fig. 15(a) offers the total primary active and reactive power waveforms (P and Q). Fig. 15(b) shows the calculated load angle per each phase.

Figs. 16(a-b) depict the post-fault operating points of the relay characteristics based on the coherence coefficients computed for the current waves for case study 3. Fig. 16(a) offers the auto-coherence coefficients (Ci_a, Ci_b, and Ci_c), and Fig. 16(b) exhibits the cross-coherence coefficients (Ci_{ab}, Ci_{bc}, and Ci_{ca}).

After the fault clearance, it is evident the following items:

- The three-phase load angles of the first SG increase rapidly and fluctuate severely due to the loss-of-synchronism condition.
- The values of the total active (P) are negative and lower than the total reactive power (Q) in some time intervals,
- All six coherence factors (Ci_a, Ci_b, Ci_c, Ci_{ab}, Ci_{bc}, and Ci_{ca}) are suddenly dropped after clearing the fault. This event occurs one time at the sample order of 7200 of the full simulation time.
- All operating points of the six coherence factors remain within the tripping zone of the proposed characteristics,
- The system enters the instability condition since the coherence deviations with respect to the ideal values are considerably increased.
- The electrical signals confirm the number times of swings (sudden changes) is one.
- In the situation of the unstable power swing, the first SG is rendered to be not synchronized with the remaining power network, leading to severe oscillations in the load angle and power flow.
- Thus, the results prove that the power swings are unstable in this case,

The results ensure that the system is stable in case 1, it is critical stable in case 2, and it is unstable in cases 3, 4, and 5.

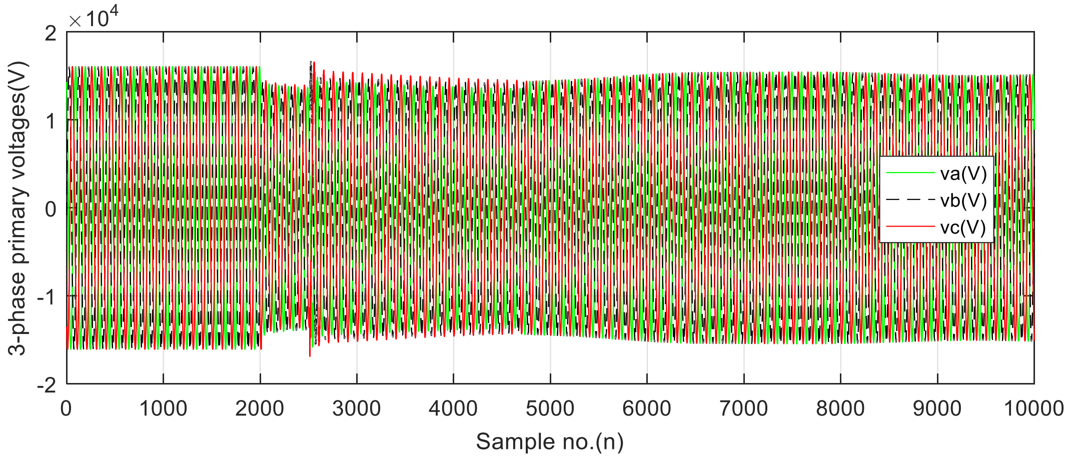

(a)

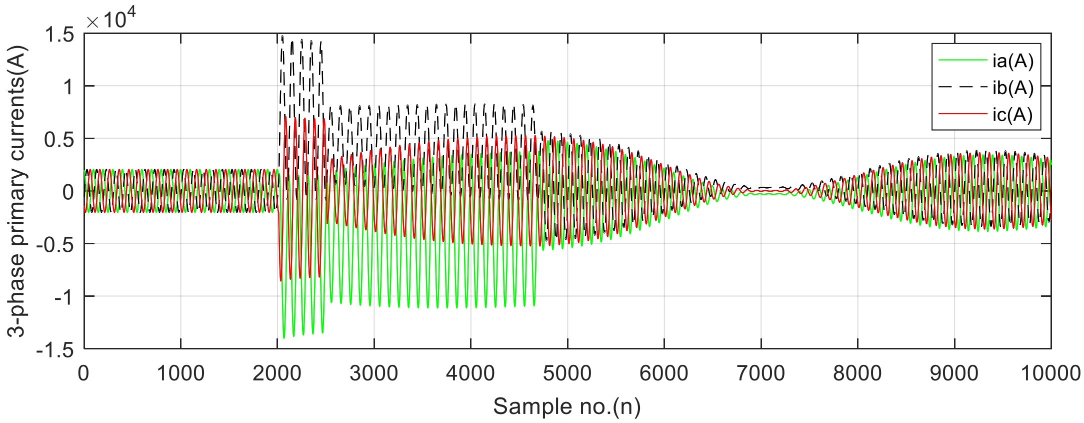

(b)

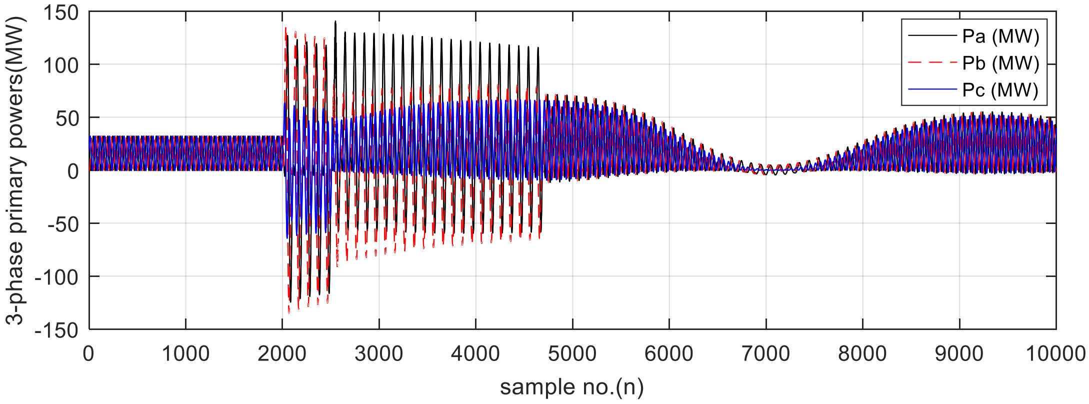

(c)

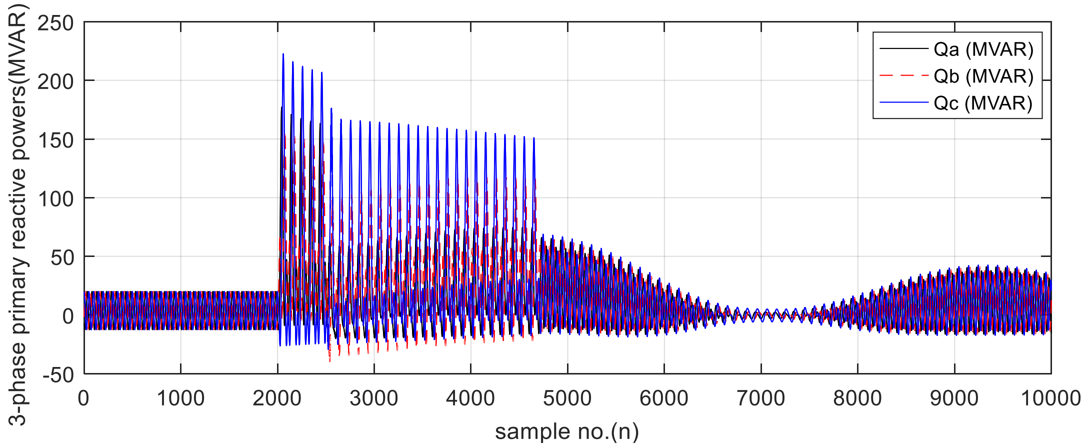

(d)

Figs. 14(a-d) The electrical waveforms taken at the first SG output for case study 3 (Unstable power swing). (a) The three-phase primary voltage waveforms (v_a, v_b and v_c), (b) The three-phase primary current waveforms (i_a, i_b and i_c), (c) The three-phase primary active power waveforms (P_a, P_b and P_c), and (d) The three-phase primary reactive power waveforms (Q_a, Q_b and Q_c).

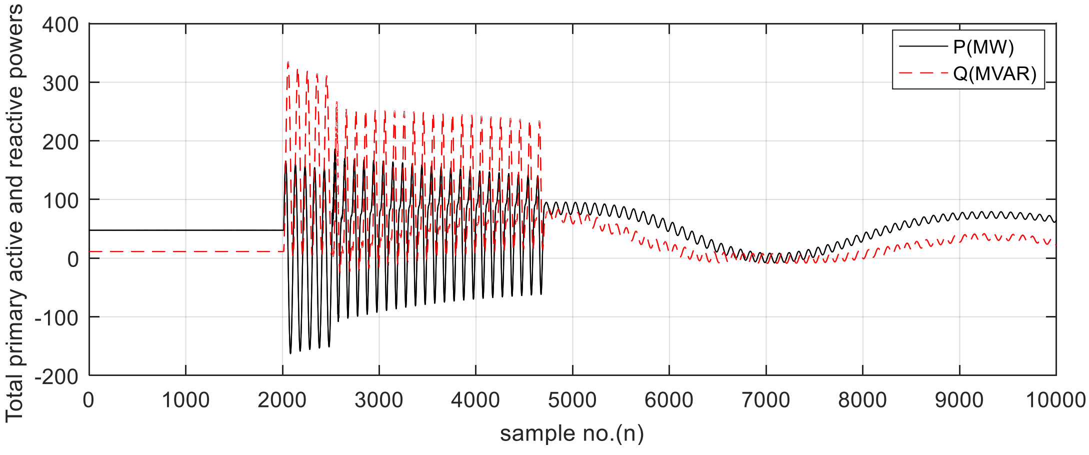

(a)

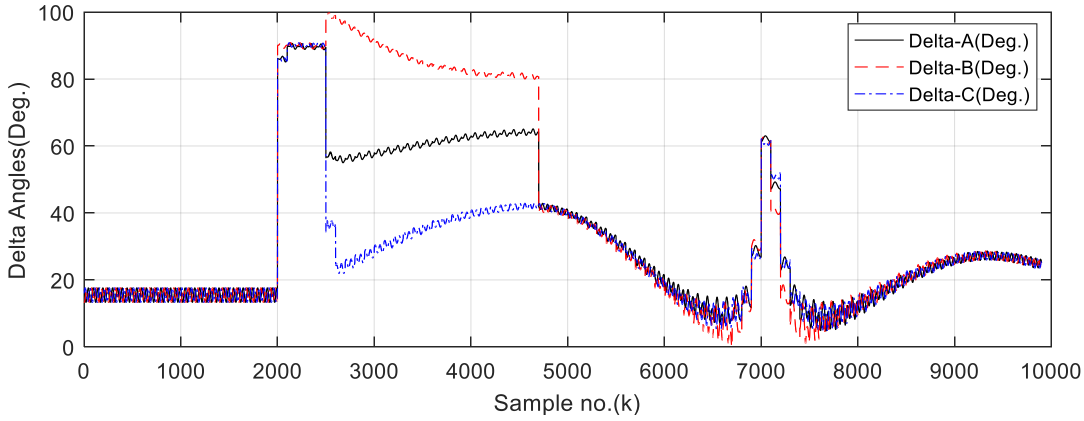

(b)

Figs. 15(a-b) The calculated variables taken at the first SG output for case study 3 (Unstable power swing), (a) The total primary active and reactive power waveforms (*P* and *Q*), and (b) The calculated load angle.

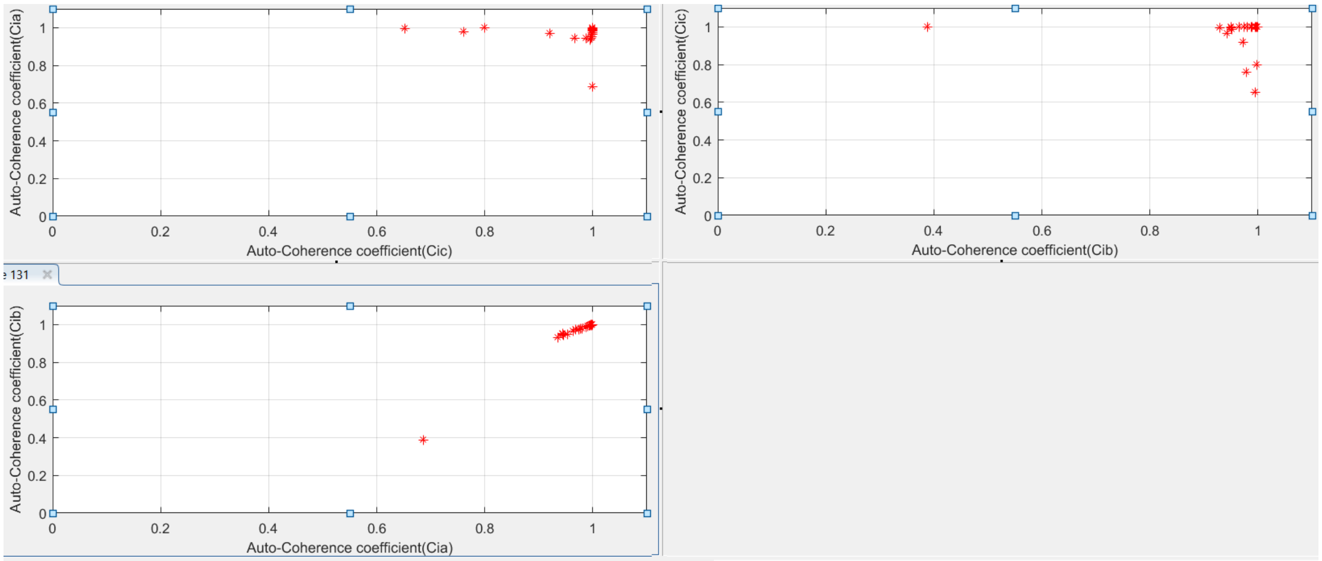

(a)

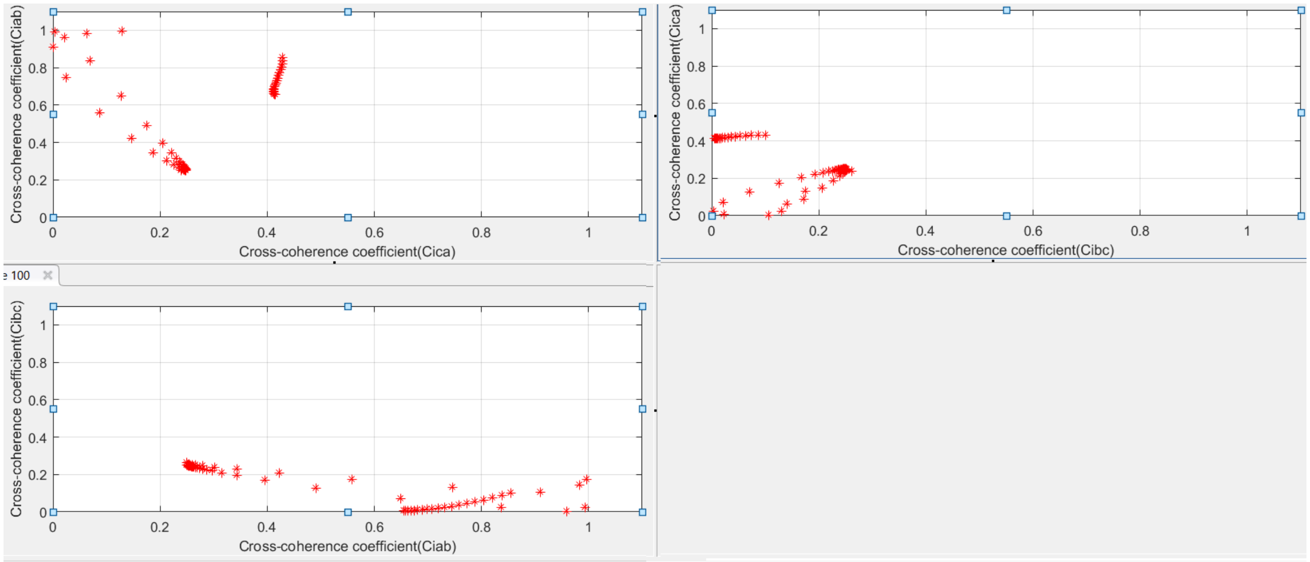

(b)

Figs. 16(a-b) The post-fault operating points of the relay characteristics based on the coherence coefficients computed for the current waves for case study 3 (Unstable power swing), (a) the auto-coherence coefficients (Ci_a, Ci_b, and Ci_c), and (b) the cross-coherence coefficients (Ci_{ab}, Ci_{bc}, and Ci_{ca}).

4.4 Case 4 Unstable power swing (δ_1= 30.0^o)

In case 4, the first SG works at a load angle (δ_1) of 30.0^o. Before and during the fault time span, it has been seen that the notes in case 4 are similar to those mentioned in case 3. Figs. 19, 20, and 21 present the simulation results for case 4. After fault clearing, it is noticed that the conditions of the unstable power swing event are verified. In case 4, the results indicate that the power swing state is unstable. The electrical signals show that there are two times of swings. After fault clearing, the sudden drops happen at the sample orders of 7700 and 9400 of the full simulation time.

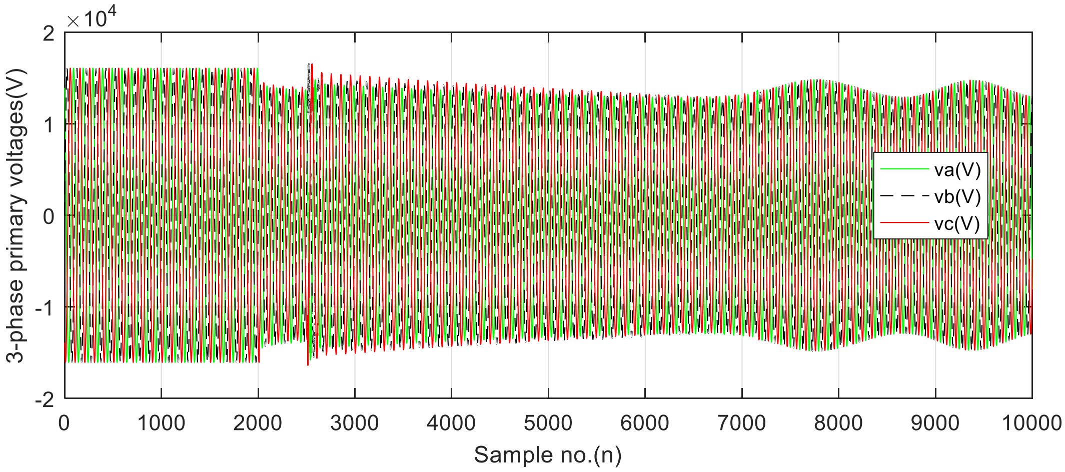

(a)

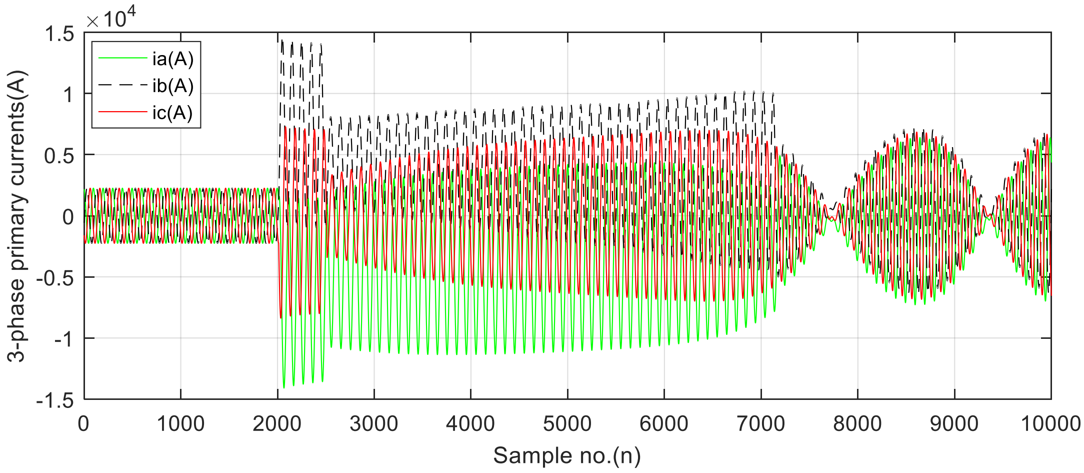

(b)

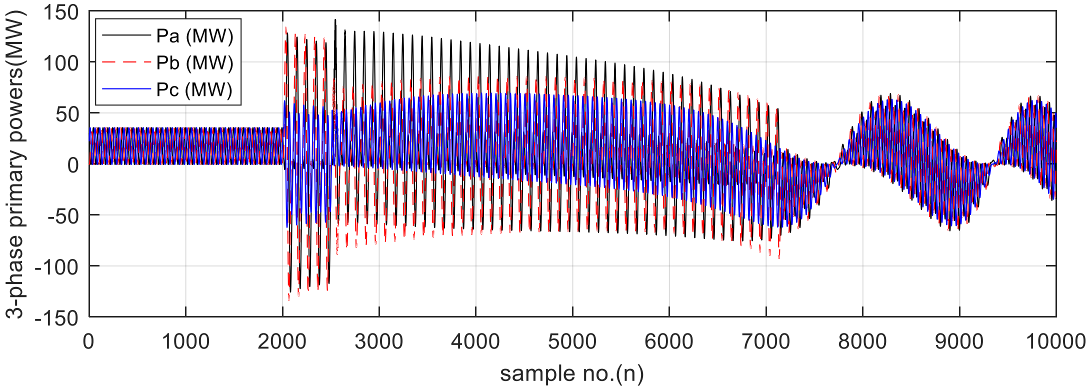

(c)

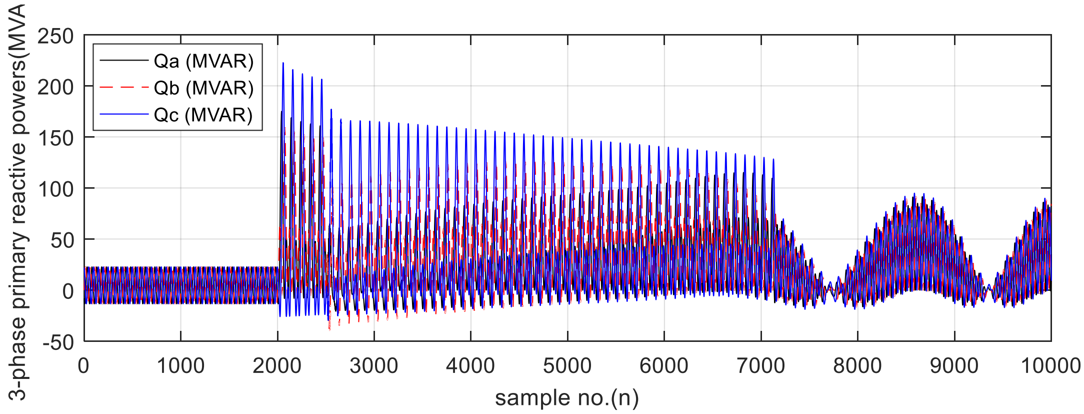

(d)

Figs. 19(a-d) The electrical waveforms taken at the first SG output for case study 4 (Unstable power swing). (a) The three-phase primary voltage waveforms (v_a, v_b and v_c), (b) The three-phase primary current waveforms (i_a, i_b and i_c), (c) The three-phase primary active power waveforms (P_a, P_b and P_c), and (d) The three-phase primary reactive power waveforms (Q_a, Q_b and Q_c).

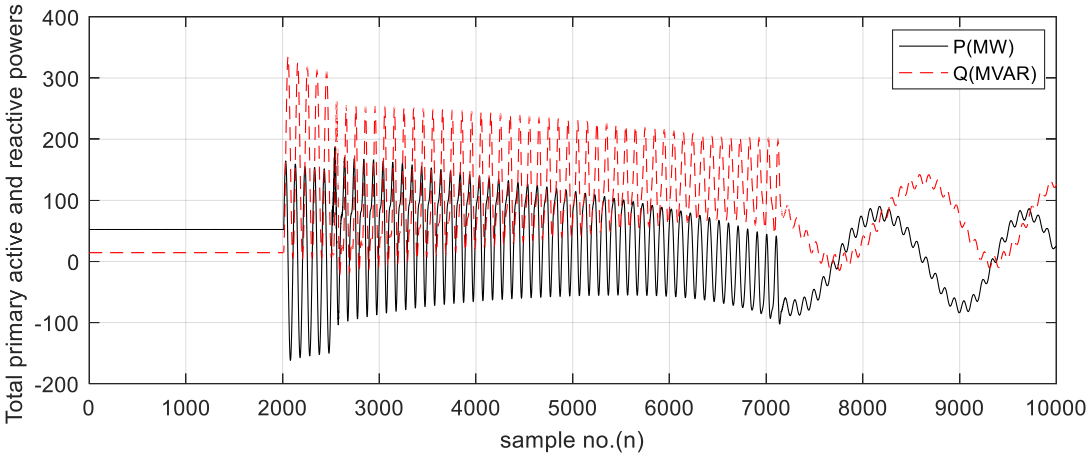

(a)

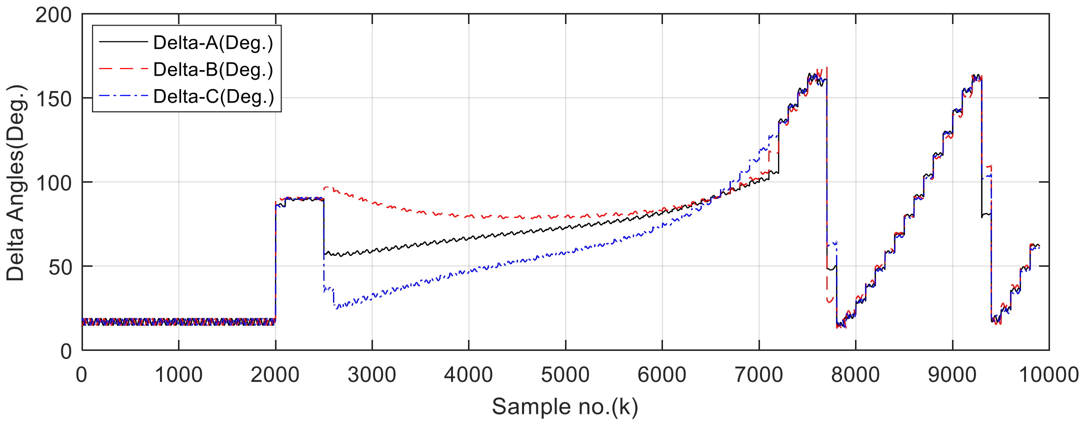

(b)

Figs. 20(a-b) The calculated variables taken at the first SG output for case study 4 (Unstable power swing), (a) The total primary active and reactive power waveforms (*P* and *Q*), and (b) The calculated load angle.

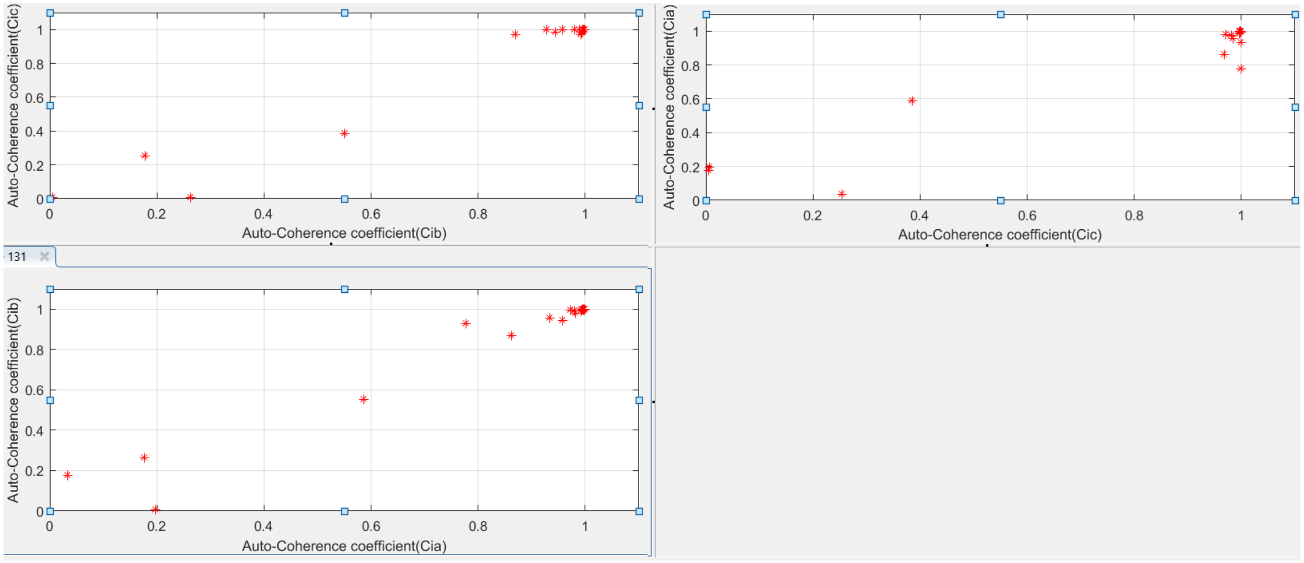

(a)

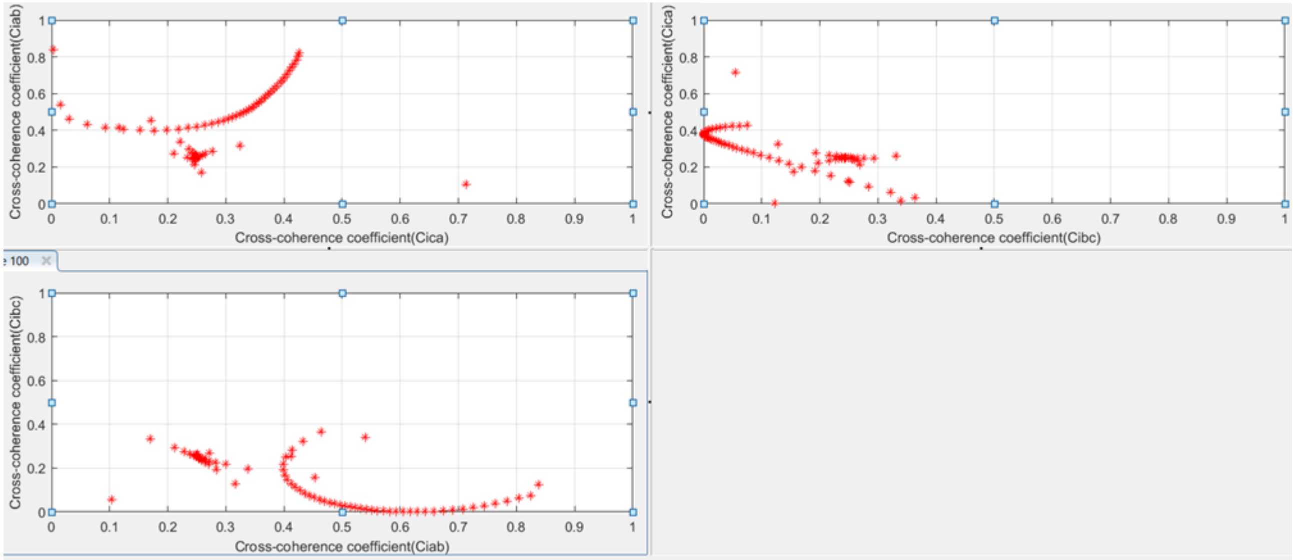

(b)

Figs. 21(a-b) The post-fault operating points of the relay characteristics based on the coherence coefficients computed for the current waves for case study 4 (Unstable power swing), (a) the auto-coherence coefficients (Ci_a, Ci_b, and Ci_c), and (b) the cross-coherence coefficients (Ci_{ab}, Ci_{bc}, and Ci_{ca}).
