# Supplementary material for: Transmission line faults detection and classification using new tripping characteristics based on statistical coherence for current measurements
Source: Sci Rep. 2025 Mar 12;15:8487. doi: 10.1038/s41598-025-87577-5 (PMC11903867; doi:10.1038/s41598-025-87577-5)
Supplement: Supplementary file 2 — Supplementary Material 2 [file 41598_2025_87577_MOESM2_ESM.pdf]

Appendix (1): Parameters' data of the power system components.

The parameter of the power system element	Data
**Machine 1 (Sending source):**	
Rated Volt-ampere	*320 MVA*
Rated line voltage	*19 kV*
Voltage phasor angle	10^0
Rated frequency	*50 Hz*
Number of poles	*2*
Neutral grounding impedance (R_n)	*0.77 Ω*
**Power Network (Receiving source):**	
Nominal line voltage	*500kV*
Voltage phasor angle	0^0
Nominal frequency	*50 Hz*
Volt-ampere short circuit	*25 GVA (* $i_{s.c}$ *= 10 kA)*
**Step-up Transformers:**	
Rated Volt-ampere	*340 MVA*
Transformation voltage ratio	*19.57 kV /500 kV*
Connection primary/secondary	*Delta/Star earthed neutral*
Primary winding impedance (Z_p)	*0.0027 + j0.184 Ω*
Secondary winding impedance (Z_s)	*0.7708 + j 61.8 Ω.*
Vector group	*YNd1*
Z%	*15%*

**Transmission Lines (1&2):**	
Positive sequence R	*0.0217 Ω /km*
Zero sequence R	*0.247 Ω /km*
Positive sequence XL	*0.302 Ω /km*
Zero sequence XL	*0.91 Ω /km*
Positive sequence 1/Xc	*3.96 μ℧ /km*
Zero sequence 1/Xc	*2.94 μ℧ /km*
Transmission line long (Km)	*200 Km*
**Aux. Load (load 1):**	
Load 1 Volt-ampere	*30 MVA at PF = 0.85 lag*
**Main Load (load 2):**	
Load 2 Volt-ampere	*25 GVA at PF = 0.85 lag*
**Current Transformer (CT):**	
CTR	*1000/1*
Rated burden	*30 VA*
Class	*5p20*
